# Structural Basis of Dual Specificity of Sinorhizobium meliloti Clr, a cAMP and cGMP Receptor Protein

**DOI:** 10.1128/mbio.03028-22

**Published:** 2023-04-05

**Authors:** Laura Werel, Neda Farmani, Elizaveta Krol, Javier Serrania, Lars-Oliver Essen, Anke Becker

**Affiliations:** a Department of Chemistry, Philipps-Universität Marburg, Marburg, Germany; b Department of Biology, Philipps-Universität Marburg, Marburg, Germany; c Center for Synthetic Microbiology (SYNMIKRO), Philipps-Universität Marburg, Marburg, Germany; Duke University School of Medicine

**Keywords:** nucleotide second messenger, CRP-like protein family, allosteric control, Clr regulon, root nodule symbiosis

## Abstract

In bacteria, the most prevalent receptor proteins of 3′,5′-cyclic AMP (cAMP) and 3′,5′-cyclic GMP (cGMP) are found among transcription factors of the Crp-Fnr superfamily. The prototypic Escherichia coli catabolite activator protein (CAP) represents the main Crp cluster of this superfamily and is known to bind cAMP and cGMP but to mediate transcription activation only in its cAMP-bound state. In contrast, both cyclic nucleotides mediate transcription activation by Sinorhizobium meliloti Clr, mapping to cluster G of Crp-like proteins. We present crystal structures of Clr-cAMP and Clr-cGMP bound to the core motif of the palindromic Clr DNA binding site (CBS). We show that both cyclic nucleotides shift ternary Clr-cNMP–CBS-DNA complexes (where cNMP is cyclic nucleotide monophosphate) to almost identical active conformations, unlike the situation known for the E. coli CAP-cNMP complex. Isothermal titration calorimetry measured similar affinities of cAMP and cGMP binding to Clr in the presence of CBS core motif DNA (equilibrium dissociation constant for cNMP (*K_D_*^cNMP^], ~7 to 11 μM). However, different affinities were determined in the absence of this DNA (*K_D_*^cGMP^, ~24 μM; *K_D_*^cAMP^, ~6 μM). Sequencing of Clr-coimmunoprecipitated DNA as well as electrophoretic mobility shift and promoter-probe assays expanded the list of experimentally proven Clr-regulated promoters and CBS. This comprehensive set of CBS features conserved nucleobases that are consistent with the sequence readout through interactions of Clr amino acid residues with these nucleobases, as revealed by the Clr–cNMP–CBS-DNA crystal structures.

## INTRODUCTION

One of the most ubiquitous nucleotide second messengers in prokaryotes and eukaryotes is 3′,5′-cyclic AMP (cAMP). It was the first second messenger to be described, initially for its role in hormone-dependent signal transduction by eukaryotes (1, 2) and later for catabolite repression in bacteria like Escherichia coli (3). Since then, a myriad of biological processes regulated by cAMP have been reported in bacteria, such as carbon metabolism, biofilm formation, type III secretion, virulence, and symbiosis (4, 5). Another cyclic mononucleotide second messenger widespread in eukaryotes is 3′,5′-cyclic GMP (cGMP) (6). Involvement of cGMP in bacterial regulation has been recognized only recently (7).

In bacteria, cyclic AMP receptor proteins (CRPs) are among the best characterized transcription factors and model for allosteric regulation (3, 8–10). Physiological effects of gene regulation by CRP-like proteins have been characterized in various bacterial species. They are highly versatile, controlling expression of more than 200 genes in E. coli and almost as many in Mycobacterium tuberculosis and Corynebacterium glutamicum (11–15).

CRPs belong to the superfamily of Crp-Fnr transcription regulators, which are composed of an N-terminal nucleotide binding domain and a C-terminal helix-turn-helix (HTH) motif (16). These domains are each conserved in eukaryotes and prokaryotes (4, 17). Upon DNA binding, the C-terminal domain of CRPs is able to interact with the bacterial RNA polymerase (RNAP) and to modulate promoter activation (18–20). Most of the CRPs act as transcription activators, while few act as repressors (6).

A phylogenetic analysis of CRP-like proteins from the Crp-Fnr superfamily grouped these proteins into a main Crp cluster, containing E. coli catabolite activator protein (CAP), and in addition suggested two associated CRP-like clusters, named F and G (16). Each of the clusters of CRP-like proteins mostly contains members from a broad distribution of proteobacterial genera, with some exceptions from other bacterial phyla. So far, all structurally characterized CRPs belong to the main Crp cluster, including E. coli CAP (21). These proteins are activated by cAMP (16). A prominent member of cluster F is *Synechocystis* sp. SyCRP1, a membrane-bound transcription factor, which is suggested to be released from the membrane through cAMP upon sensing of inorganic carbon (22). CgrA from Rhodospirillum centenum and Clr from Sinorhizobium meliloti, two functionally studied CRPs, can be assigned to cluster G by applying an approach described previously (16). CgrA appeared to be solely activated by cGMP with very low cAMP-binding affinity, whereas Clr is distinguished from any of the other known CRP homologues by its ability to be activated by both cAMP and cGMP (23, 24). cGMP binding has been described for E. coli CAP as well, albeit without stimulating DNA binding (25).

The soil-dwelling alphaproteobacterium S. meliloti is capable of fixing atmospheric nitrogen in a symbiotic relationship with leguminous plants of the genera *Medicago*, *Melilotus*, and *Trigonella*. The symbiotic program involves root hair infections and formation of root nodules, harboring the nitrogen-fixing bacteria (26, 27). The S. meliloti Rm1021 and Rm2011 genomes encode 13 proteins belonging to the CRP-Fnr superfamily, including the cAMP-independent FixK regulators of microoxic respiration and nitrogen fixation genes (3) and cAMP- and cGMP-controlled Clr (28). Eight of those are homologous to CRP-like proteins. Activation of Clr both by cAMP and cGMP is especially interesting given that these S. meliloti genomes contain an exceptionally high number of 28 putative adenylate cyclase/guanylate cyclase (AC/GC) genes (29). Clr and the AC/GCs, CyaD1, CyaD2, and CyaK, are implicated in repression of secondary infections of Medicago sativa roots (5). Moreover, Clr overexpression resulted in reduced swimming motility and increased succinoglycan production, which are traits relevant at early stages of the root nodule symbiosis (24).

In our study, we present crystal structures of Clr-cNMP-DNA complexes (where cNMP is cyclic nucleotide monophosphate), which to our knowledge is the first report of a cluster G CRP structural analysis. Our analysis unravels the molecular basis of Clr specificity for cAMP and cGMP. Furthermore, we expand the set of experimentally proven Clr-regulated promoters and Clr DNA binding site (CBS). These feature conserved nucleobases which are in accordance with the sequence readout revealed by the Clr–cNMP–CBS-DNA crystal structures.

## RESULTS

### ChIP-seq-assisted screening for candidate Clr binding sites.

**FIG 1:**
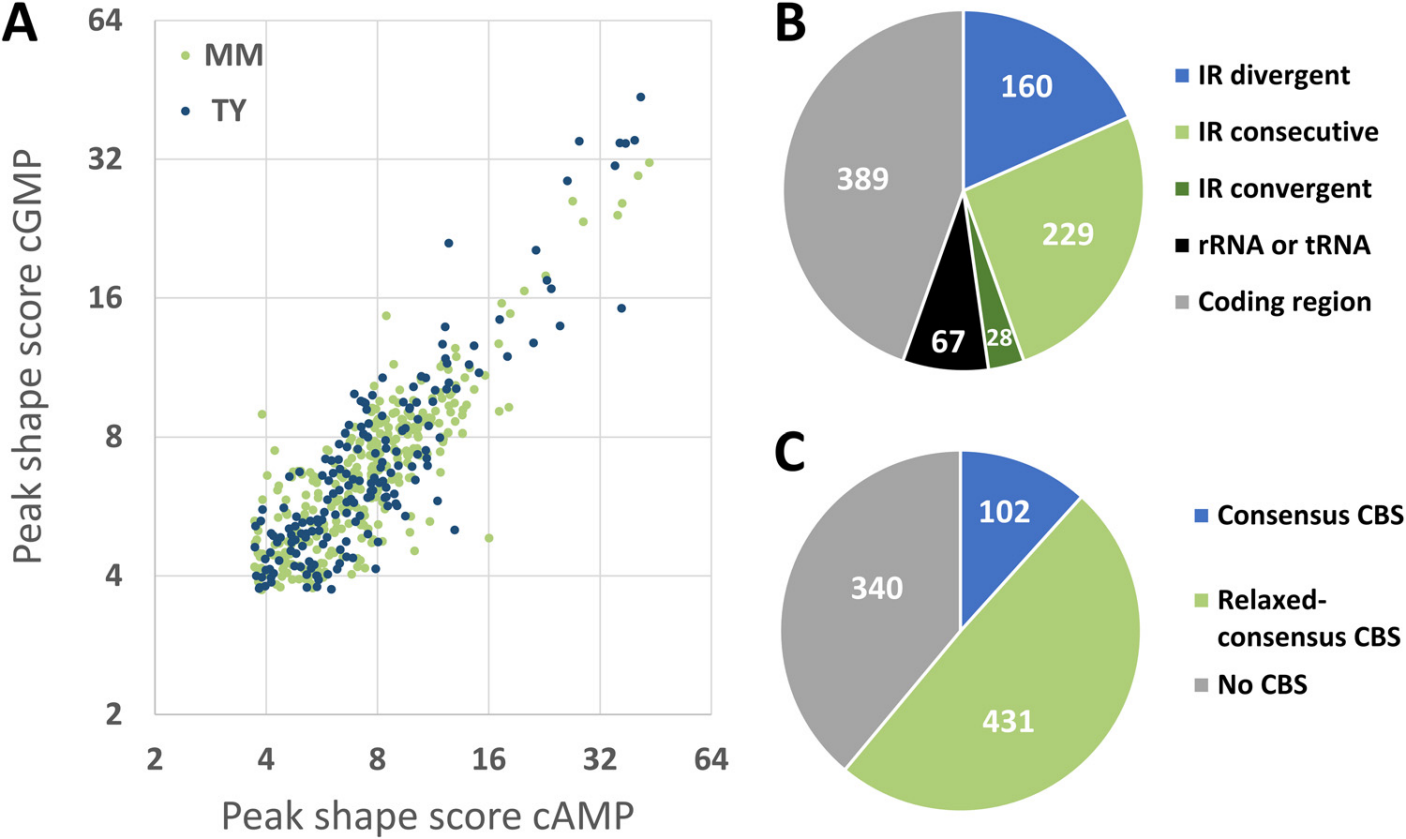
ChIP-seq-assisted screening for Clr-cAMP and Clr-cGMP binding sites. (A) Correlation between peak shape score values of ChIP-seq peaks detected in samples of cells grown in TY or MM medium, both supplemented with either cAMP or cGMP. (B) Location of Clr ChIP-seq peaks within genomic features. IR, intergenic region; IR divergent, IR between two genes transcribed in divergent directions; IR consecutive, IR between two genes transcribed in the same direction; IR convergent, IR between two genes transcribed in converging directions; rRNA or tRNA, rRNA or tRNA encoding region; coding region, protein coding region. (C) Presence of putative Clr binding sites (CBSs) in the vicinity of ChIP-seq peaks. Consensus CBS, GTNNCNNNNGNNAC. Relaxed-consensus CBS, one mismatch with the consensus was allowed.

To identify Clr-cAMP and Clr-cGMP binding sites genome-wide in the DNA of S. meliloti Rm2011, we used chromatin immunoprecipitation coupled with deep sequencing (ChIP-seq). For this purpose, we first generated plasmid pWBT-Clr-CF and introduced it into S. meliloti strain Rm2011 Δ*clr*. pWBT-Clr-CF carries IPTG (isopropyl-β-d-thiogalactopyranoside)-inducible *clr-3xflag*, encoding Clr C-terminally fused to a 3×FLAG tag. *In vivo* functionality of Clr-3×FLAG was confirmed by its ability to activate plasmid-borne *egfp* reporter gene fusions to promoters of the previously identified target genes *SMc02178* and *SMb20906* (24) in Rm2011 Δ*clr* in the presence of externally added cAMP (Fig. S1). For anti-FLAG antibody-mediated chromatin immunoprecipitation, Rm2011 Δ*clr* carrying pWBT-Clr-CF was cultivated in tryptone-yeast extract (TY) complex medium or morpholinepropanesulfonic acid (MOPS)-buffered minimal medium (MM) and supplemented with either cAMP or cGMP. Deep sequencing of DNA enriched for Clr-bound regions and control samples representing sheared DNA isolated from cell lysates prior to immunoprecipitation yielded 3.3 to 3.9 million reads per sample (see Table S1A in the supplemental material). ChIP-seq data were processed using the CLC Genomics Workbench to determine peak locations and peak shape score values, which reflect enrichment of the respective regions (Table S1B to E). Consolidation of our data obtained for the four samples yielded 873 distinct ChIP-enriched genomic regions, further referred to as ChIP peaks (Table S1F). Consistent with our previous observation that both cAMP and cGMP promote Clr binding to DNA (24), 40 out of 50 top-score ChIP-enriched regions were detected in samples from cultures supplemented with either of the two cNMPs (Table S1F). Moreover, peak shape score values correlated well between cAMP and cGMP samples (correlation factors of 0.92 and 0.91 in TY and MM media, respectively) (Fig. 1A).

10.1128/mbio.03028-22.1 FIG S1 Functionality of Clr-CF. Download FIG S1, PDF file, 0.2 MB. Copyright © 2023 Werel et al. 2023 Werel et al. https://creativecommons.org/licenses/by/4.0/ This content is distributed under the terms of the Creative Commons Attribution 4.0 International license.

10.1128/mbio.03028-22.6 TABLE S1 ChIP-seq data and analyses. Download Table S1, XLSX file, 1.0 MB. Copyright © 2023 Werel et al. 2023 Werel et al. https://creativecommons.org/licenses/by/4.0/ This content is distributed under the terms of the Creative Commons Attribution 4.0 International license.

Out of 417 ChIP peaks identified within intergenic regions, 160 were located between two divergently transcribed genes, 229 between two genes transcribed in the same direction, and 28 between two convergently transcribed genes. The remaining ChIP peaks were situated within rRNA or tRNA genes and protein coding regions (Fig. 1B). To search for Clr binding sites (CBSs) in ChIP-enriched regions, we extracted 400-bp DNA sequences surrounding the peak centers. Using the FIMO online tool (30), these regions were scanned for the core Clr binding site motif GTNNCNNNNGNNAC. This core motif was derived from the consensus sequence HGTYHCNNNNGRWACA, which represents six experimentally verified CBSs (24). Sequences matching the core motif were found in 102 ChIP-enriched regions, corresponding to a total of 107 putative CBSs, of which 87 were located in intergenic regions (Fig. 1C; Table S1G). A FIMO search with relaxed settings (*P* value, 0.001) identified additional 693 putative CBSs with one mismatch in the core motif (relaxed-consensus CBSs) in 431 ChIP-enriched regions (Table S1H). Of these, 89 putative CBSs were found in 51 ChIP-enriched regions, which already contained a strict consensus sequence.

As expected, all previously characterized CBS sequences in the promoter regions of *SMc02178*, *SMc00653*, *SMc01136*, *SMc04190*, *cyaF2*, and *SMb20906* (5, 24) were found within the ChIP-enriched regions. Among genes adjacent to ChIP peak-containing intergenic regions, 35 genes were found (Table S1I) that were previously identified in transcriptome experiments to be upregulated upon overexpression of *clr* or adenylate cyclase gene *cyaJ* (24, 31). Twenty-five of these genes contained putative consensus and/or relaxed-consensus CBSs in their upstream noncoding regions. The remaining 10 genes lacked putative CBSs in their vicinity. Remarkably, these 10 genes were only detected as being transcriptionally activated upon *cyaJ*, not *clr*, overexpression.

### Clr-cAMP and Clr-cGMP specifically bind DNA target sequences *in vitro*.

**FIG 2:**
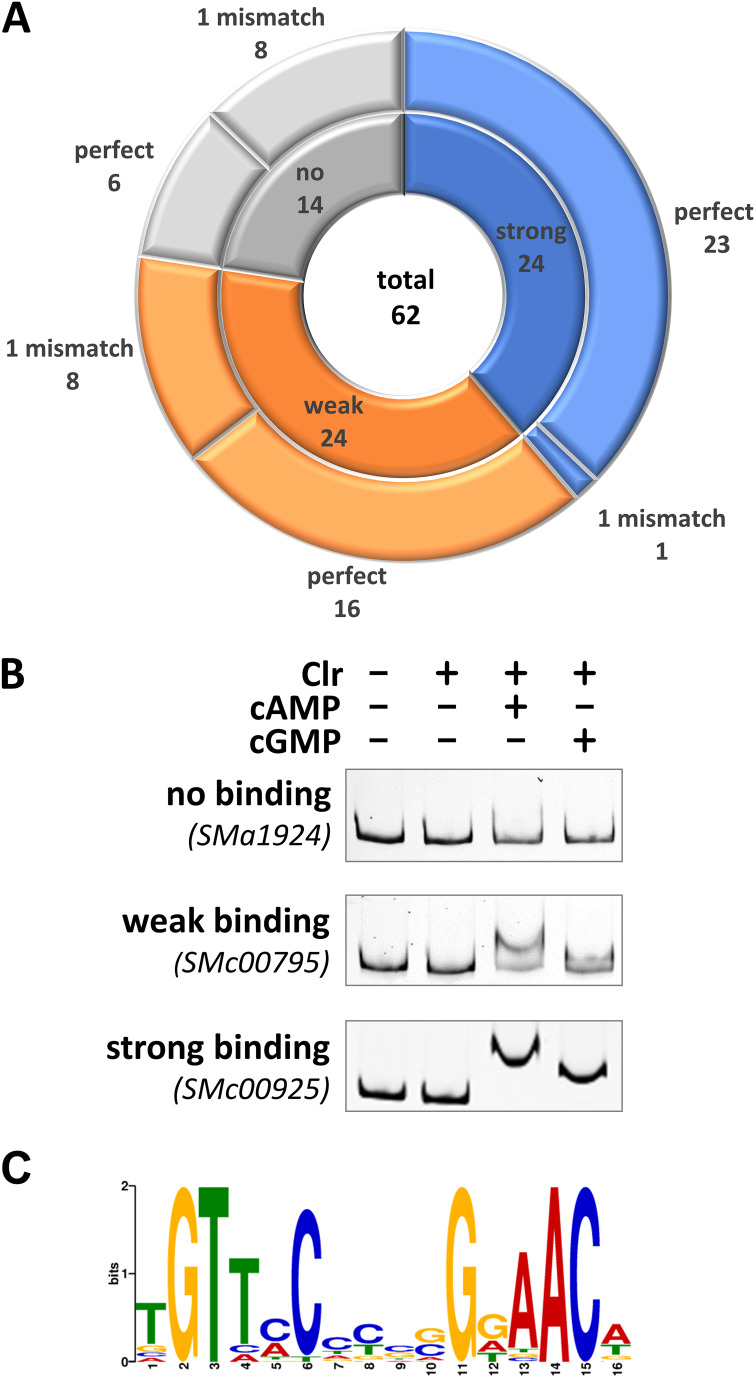
EMSA-based subclassification of CBS. All DNA fragments used for EMSAs contained at least one putative CBS matching the consensus binding site sequence either perfectly or with one mismatch. (A) EMSAs were subclassified into strong (blue), weak (orange), and no binding (gray). Numbers of perfect and imperfect binding sites are indicated for each subclass. (B) Representative EMSAs are shown for each subclass. These were performed with upstream regions of *SMa1924* (no binding), *SMc00795* (weak binding), and *SMc00925* (strong binding), which each contained a perfect-match binding site. (C) MEME logo of 24 Clr binding sites, conferring strong Clr binding in EMSAs.

**FIG 3:**
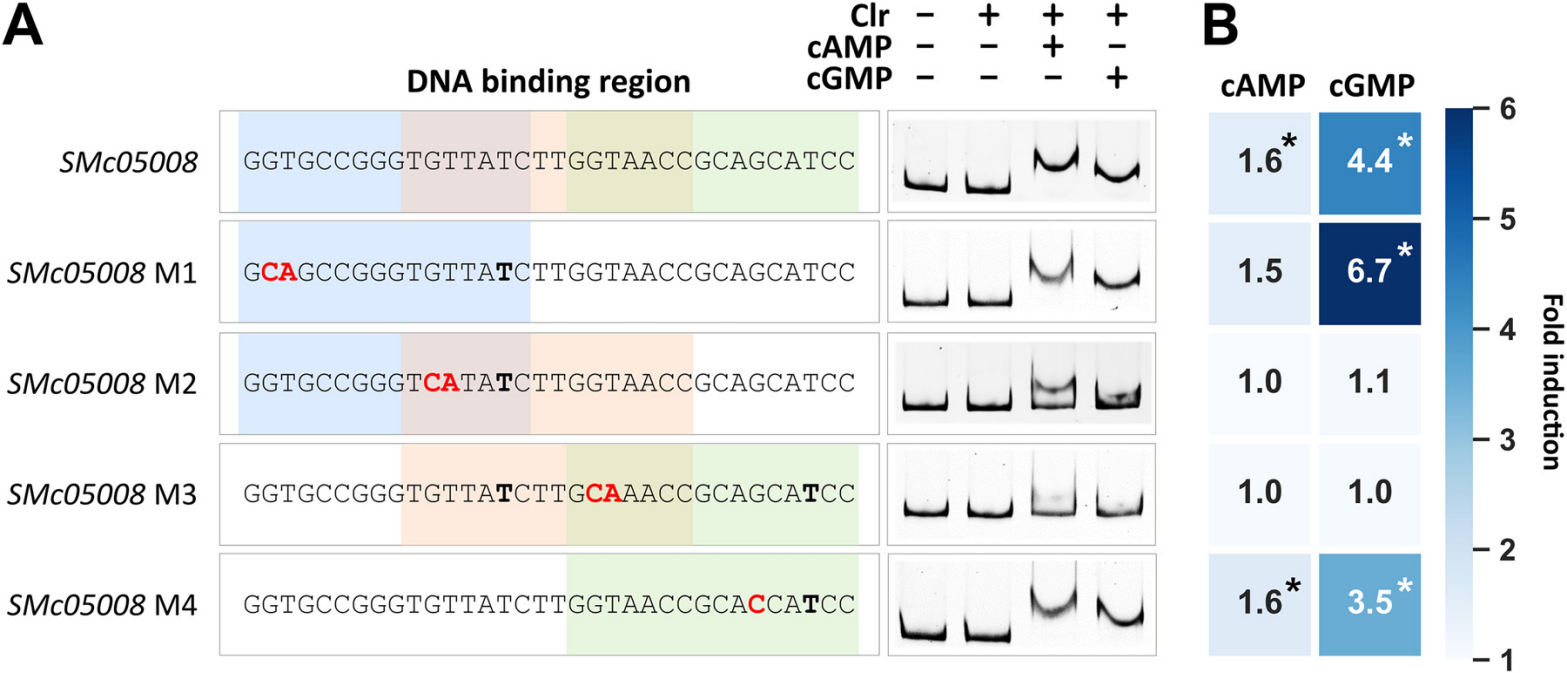
DNA binding of Clr-cAMP and Clr-cGMP at the *SMc05008* promoter. (A) The promoter region of *SMc05008* has three possible DNA binding sites (colored), with one mismatch each. Mutations indicated in red were introduced at four different positions (M1 to M4). Nucleotides not matching the consensus Clr binding sequence are shown in bold. (B) A heat map shows the cAMP- or cGMP-induced fold change of EGFP-mediated fluorescence compared to that of noninducing conditions (no cNMP added). Asterisks indicate significant changes in the EGFP-mediated fluorescence (Table S2).

To characterize putative CBS sequences identified within ChIP-seq-enriched DNA regions and located in upstream noncoding regions, we performed electrophoretic mobility shift assays (EMSAs) for 62 genomic regions (Fig. 2A; Fig. S2A to D). The corresponding DNA fragments that produced a complete electrophoretic mobility shift in the presence of Clr and cAMP were defined as regions with a strong CBS, whereas DNA fragments that were only partially shifted were defined as containing a weak CBS (Fig. 2B). Of the 62 CBS candidates tested, 24 were classified as strong and 24 as weak CBSs, while the remaining failed to mediate a detectable band shift in the EMSAs (Fig. 2A; Fig. S2A to D). Furthermore, Clr-cAMP produced stronger band shifts than Clr-cGMP. Not all DNA regions containing putative CBSs matching the consensus produced strong band shifts, which indicates that nonconserved nucleotides within the CBS and its sequence environment might contribute to Clr-cNMP binding. For strong binding sites, we noted that the left and right halves of the palindrome tended to be enriched for pyrimidine (C/T) and purine (G/A) base nucleotides, respectively (Fig. 2C; Table S1J). Conversely, putative CBSs mediating a weak or no band shift lacked a clear nucleotide preference at the nonconserved positions. Almost all DNA fragments causing a strong band shift included a CBS sequence that matched the core motif. A notable exception is the DNA fragment covering the *SMc05008* promoter region, as this region contains three overlapping putative CBSs, each with one mismatch in the core motif (Fig. 3A).

10.1128/mbio.03028-22.2 FIG S2 EMSA shift patterns of all putative Clr binding sites and promoter-probe measurements in S. meliloti. Download FIG S2, PDF file, 0.7 MB. Copyright © 2023 Werel et al. 2023 Werel et al. https://creativecommons.org/licenses/by/4.0/ This content is distributed under the terms of the Creative Commons Attribution 4.0 International license.

10.1128/mbio.03028-22.7 TABLE S2 Clr-dependent promoter-probe activation. Download Table S2, XLSX file, 0.7 MB. Copyright © 2023 Werel et al. 2023 Werel et al. https://creativecommons.org/licenses/by/4.0/ This content is distributed under the terms of the Creative Commons Attribution 4.0 International license.

To verify the CBS location within DNA regions that showed strong Clr-cNMP binding, we exchanged the conserved GT in the left half of the palindrome to CA. This modification dramatically reduced Clr-cNMP binding, thus confirming the predicted CBS within these regions (Fig. S2A to D). Of the three overlapping putative CBSs in the *SMc05008* promoter region, only mutations in conserved nucleotide positions of the overlapping putative second and third CBSs inhibited Clr-cNMP binding (Fig. 3A). Band shift assays with synthetic DNA fragments containing either one of the putative CBSs identified in the *SMc05008* promoter region suggested that only the second putative CBS conferred Clr-cNMP binding (Fig. S2D). This indicates that a C→T transition at position 6 of the CBS core motif is not detrimental *per se* for Clr-cNMP binding to the DNA.

### Target promoters are regulated by both Clr-cAMP and Clr-cGMP *in vivo*.

All tested CBS candidates were further investigated for Clr-cNMP-dependent promoter activation *in vivo*. In cases where the CBS was located in noncoding regions between two opposingly transcribed genes, both putative promoter regions were tested. Plasmid-borne copies of promoter regions followed by up to 75 bp of the downstream coding regions or noncoding RNA genes fused to *egfp* were introduced into S. meliloti or E. coli for promoter activity assays.

**FIG 4:**
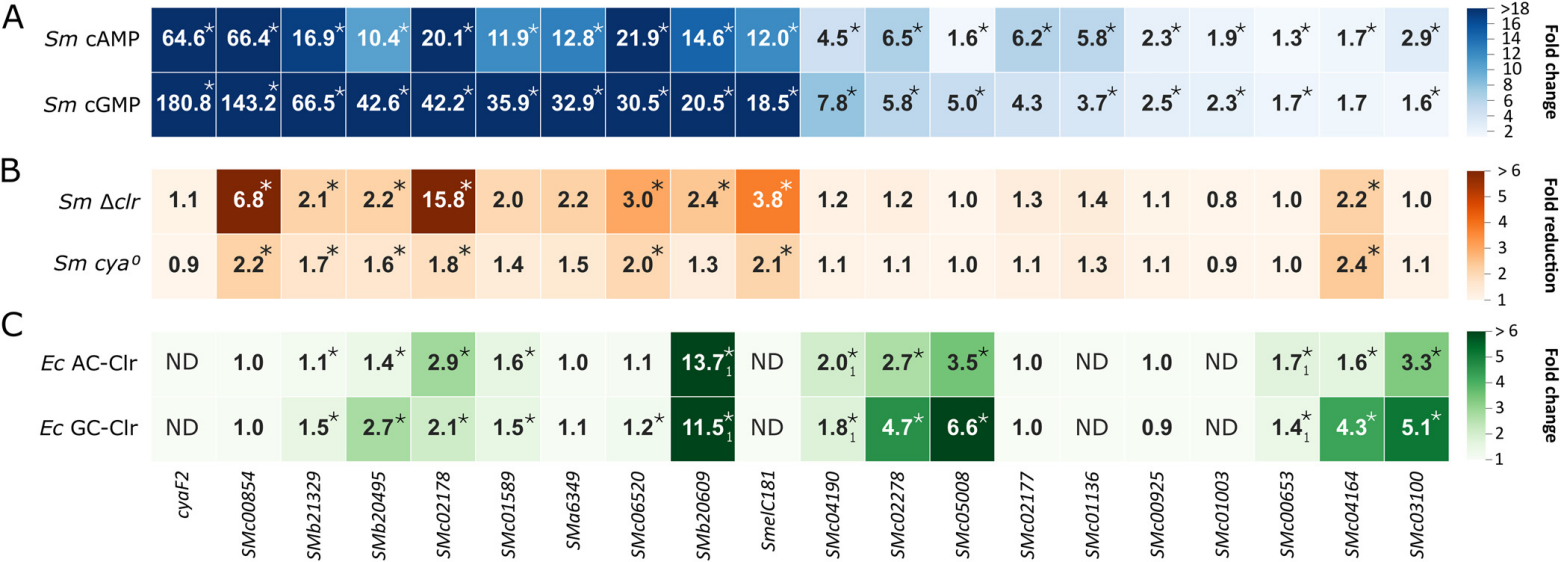
Promoter-probe measurements in different hosts. (A) Fold change of EGFP fluorescence signal mediated by promoter-probe constructs in S. meliloti Rm2011 (*Sm*) in cultures supplemented with cAMP or cGMP. Ratios derive from relative fluorescent units (RFU) of cNMP-induced against uninduced cultures. (B) Fold reduction in EGFP fluorescence signal mediated by promoter-probe constructs in S. meliloti Rm2011 Δ*clr* and Rm2011 *cya^0^* compared to that of Rm2011 wild type. Asterisks indicate statistically significant reductions in fluorescence (*P* < 0.05) in combination with a wild-type RFU threshold of ≥80. (C) Fold change of EGFP fluorescence signal mediated by promoter-probe constructs in E. coli BTH101 (*Ec*) upon induced production of an AC (CyaG1 of S. meliloti) or a GC (Cya2 of *Synechocystis* sp.) together with Clr (S. meliloti) in LB. Ratios shown derive from fluorescence units of strains under AC/GC-Clr production conditions relative to those of control strains producing the AC or GC but not Clr. Subscript 1 indicates values derived from reference 24. ND, data not determined. Asterisks indicate significant changes in the EGFP-mediated fluorescence (Tables S2 to S4).

Reporter construct-mediated fluorescence was determined in S. meliloti Rm2011 wild type upon addition of either cAMP or cGMP (Fig. 4A; Fig. S2E and Table S2). This analysis confirmed Clr-cNMP-dependent activation of 20 promoters by at least 1.5-fold either with cAMP or cGMP added. These included only promoter regions that showed a strong band shift in the EMSAs. To estimate the effect of CBS location relative to the transcription start site (TSS) on promoter activation, we gathered S. meliloti TSS positions, determined previously in Rm2011 and Rm1021 wild-type strains (32, 33) and Rm1021 derivative RFF625c lacking all *ecf* sigma factors (34), and determined the CBS-TSS distances (Table S1G and H). For most promoters activated by Clr-cNMPs, CBS-TSS distances of −42 to −76 were found (Fig. S2F). These promoters are thus comparable to CAP-dependent class I and class II promoters in E. coli (35–37). In E. coli, these promoters are subdivided into three classes, i.e., promoters with a CAP binding site roughly 60 to 90 bp upstream of the TSS (class I), close to the −35 element (class II), and at position −91 or further upstream (class III), with the latter requiring additional binding of another transcription regulator closer to the promoter region for activation (35–37).

As expected, GT→CA mutation of the first two nucleotides of the core CBS motif abolished or substantially decreased Clr-cNMP-dependent promoter activation (Fig. S2E and Table S2). Regarding the three overlapping CBSs in the *SMc05008* promoter region, only nucleotide exchanges that diminished Clr-cNMP binding in the EMSA also abolished promoter activation by Clr-cNMP (Fig. 3B).

For the majority of CBS-containing promoter fragments tested, induction by cGMP was up to four times higher than that by cAMP. In total, we identified 11 novel Clr-cNMP-activated genes in addition to nine Clr-cNMP-activated genes reported previously (24, 31). The majority of the 20 confirmed Clr-cNMP-regulated genes encode hypothetical proteins, and, notably, 8 of the 20 genes encode small proteins of less than 100 amino acids. Ten Clr-cNMP-activated genes encode proteins with an N-terminal signal peptide. Moreover, Clr-cNMP was found to directly induce transcription of *SMelC181* encoding a small noncoding RNA of unknown function (Fig. 4A and C). This RNA was previously identified in a transcriptome sequencing (RNA-seq) study (38).

To evaluate promoter activation by Clr dependent on endogenously produced cNMPs, we compared the promoter-probe constructs conferring enhanced green fluorescent protein (EGFP) fluorescence between Rm2011 and its derivatives lacking either all annotated AC/GC genes (*cya^0^*) or *clr* (Fig. 4B; Table S3). To generate the *cya^0^* strain, all 28 annotated class III AC/GC-encoding genes (29) were sequentially deleted from the Rm2011 genome. The strains were grown in medium without added cNMPs. Under these conditions, eight and seven of the 20 promoter-probe constructs mediated at least 1.5-fold (*P* < 0.05) reduced fluorescence levels in the Δ*clr* and *cya^0^* strains, respectively, compared to the wild type (Fig. 4B; Table S3). Lack of the *clr* gene resulted in a much stronger reduction in the promoter activities of *SMc00854*, *SMc02178*, and the sRNA gene *SMelC181* than absence of the 28 AC/GC genes.

10.1128/mbio.03028-22.8 TABLE S3 EGFP-derived fluorescence fold reduction in S. meliloti Δ*clr* and *cya^0^*. Download Table S3, XLSX file, 0.06 MB. Copyright © 2023 Werel et al. 2023 Werel et al. https://creativecommons.org/licenses/by/4.0/ This content is distributed under the terms of the Creative Commons Attribution 4.0 International license.

Furthermore, Clr-cNMP-mediated transcription activation of, overall, 17 promoter regions with confirmed and putative Clr-binding motifs was analyzed in E. coli as a heterologous host (Fig. 4C; Table S4). We used E. coli strain BTH101, which is unable to produce cAMP. This strain was equipped with plasmid-borne *clr*, coexpressed with either the S. meliloti adenylate cyclase gene *cyaG1* or the *Synechocystis* sp. guanylate cyclase gene *cya2* under the control of an IPTG-inducible promoter. Promoter-reporter constructs were introduced on a second plasmid. We determined previously that in E. coli BTH101, cAMP/cGMP production alone was insufficient to activate the S. meliloti Clr-cNMP target promoters, and therefore E. coli CRP did not replace Clr (24). Out of the 17 promoters tested, eight were activated by Clr-cNMP in E. coli by at least 1.5-fold either by cAMP or cGMP production. Including the three previously described candidates, *SMb20906*, *SMc00653*, and *SMc04190* (24), a total of 11 promoters were activated by Clr-cNMP in E. coli. Promoters that were not clearly activated by Clr-cNMP in S. meliloti Rm2011 also showed no activation in E. coli BTH101 (Tables S2 and S4). In contrast, five promoters (*SMc00854*, *SMa6349*, *SMc06520*, *SMc02177*, and *SMc00925*) that were activated in S. meliloti Rm2011 were not activated in E. coli BTH101, suggesting that these promoters might require additional S. meliloti-specific factors for Clr-cNMP-mediated activation.

10.1128/mbio.03028-22.9 TABLE S4 Clr-cNMP-dependent promoter-probe activation in E. coli BTH101. Download Table S4, XLSX file, 0.1 MB. Copyright © 2023 Werel et al. 2023 Werel et al. https://creativecommons.org/licenses/by/4.0/ This content is distributed under the terms of the Creative Commons Attribution 4.0 International license.

### Clr affinity for cGMP increases in the presence of DNA.

**FIG 5:**
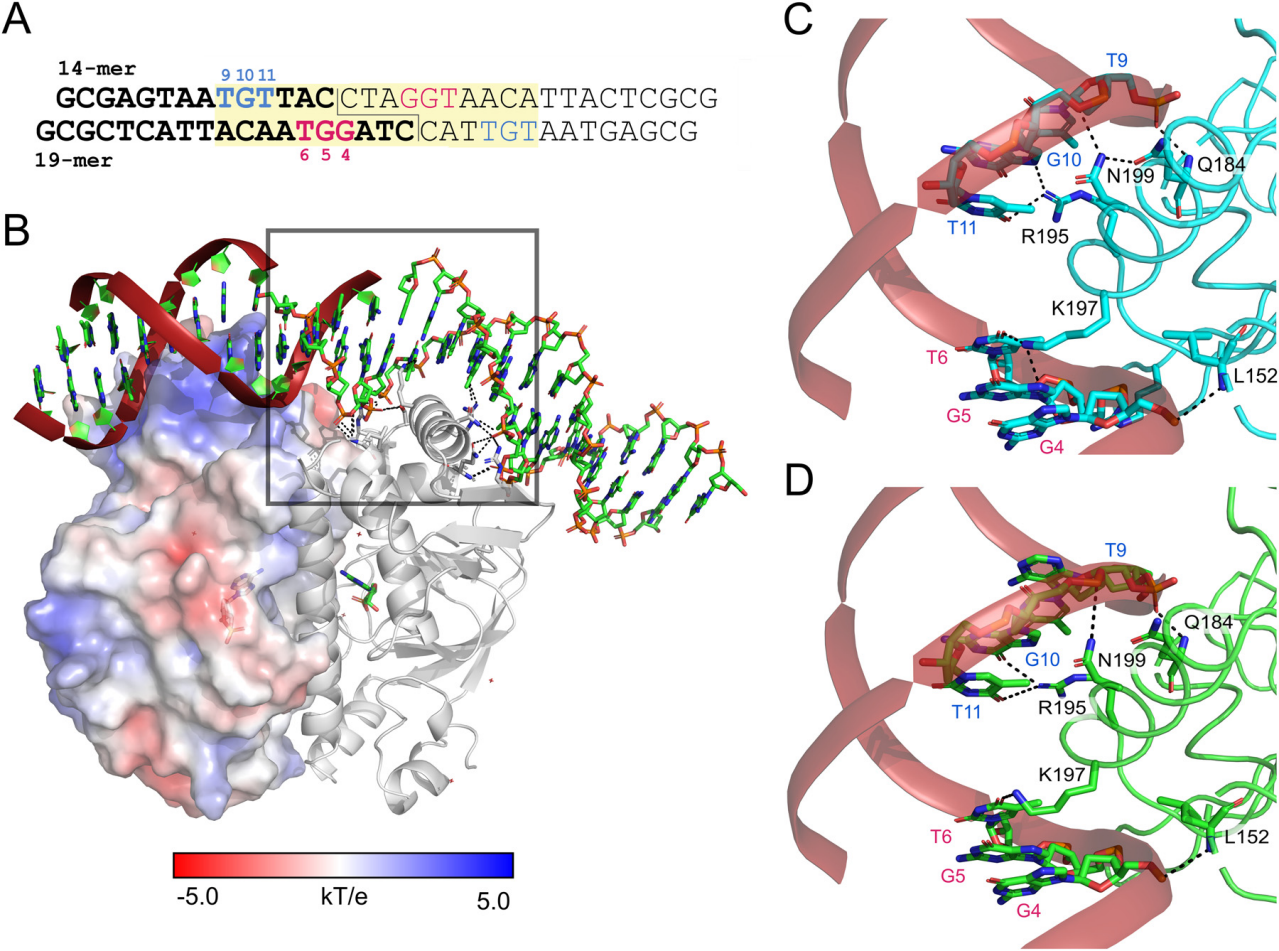
Interaction of Clr with its DNA recognition site. (A) Synthetic DNA fragment containing binding target sequence for Clr, used in ITC and crystallization. It is composed of two 14-mer and two 19-mer oligonucleotides. The two identical double-stranded fragments are shown in bold (left arm) and regular font (right arm). They are joined by a 4-bp sticky end, located right above the subunit interface. The design was based on the consensus motif GTNNCNNNNGNNAC. The 16-bp palindrome sequence containing the Clr binding consensus motif is highlighted in yellow. The nucleotide numbering is in the 5′-to-3′ orientation. (B) The surface of the helix-turn-helix DNA binding domain is positively charged to interact with the phosphate backbone. Helix α8 is inserted into the major groove and provides base readout via direct hydrogen bonding interactions. The staggered single-strand breaks above the subunit interface facilitate DNA kinking of about 40°. The 19-mer oligonucleotides belong to chains C and E, and the 14-mer oligonucleotides belong to chains D and F of the structure, respectively. (C and D) Direct interactions of Clr with the DNA major groove upon cAMP (C) and cGMP (D) binding. The DNA bases involved in the interaction are highly conserved. The color coding and the numbering of the DNA bases correspond to those shown in panel A.

As EMSA and promoter activation studies suggested cNMP-inducible binding of CBS-DNA by Clr, we further analyzed the binding behavior of Clr using isothermal titration calorimetry (ITC) (Fig. S3). A synthetic 33-nucleotide DNA sequence was designed based on the core CBS motif as a binding target for Clr. For ITC assays and subsequently for crystallization experiments (see below), this sequence was split into two identical synthetic double-stranded oligonucleotides, which dimerize via their palindromic sticky ends (Fig. 5A). The obtained thermograms are consistent with a simple model assuming one DNA binding site per Clr-cAMP complex. A similar behavior has been described for E. coli CAP (18, 39, 40) and M. tuberculosis CRP (40). The “active” form of CAP is represented by a CRP dimer-cAMP_2_ complex (39, 41). The secondary binding site proposed for E. coli CAP and M. tuberculosis CRP located in the C-terminal HTH DNA binding domain is not conserved in Clr, and no binding could be observed in ITC, hydrogen/deuterium exchange mass spectrometry (HDX-MS), or crystallographic experiments (42). As the affinity for the secondary binding site is considerably above physiological concentrations, its biological relevance has recently been dismissed (9, 41, 43, 44).

10.1128/mbio.03028-22.3 FIG S3 Raw and integrated ITC thermograms. Download FIG S3, PDF file, 0.5 MB. Copyright © 2023 Werel et al. 2023 Werel et al. https://creativecommons.org/licenses/by/4.0/ This content is distributed under the terms of the Creative Commons Attribution 4.0 International license.

The affinity of cAMP binding itself by Clr is mostly not affected by the presence of cognate CBS-DNA with equilibrium dissociation constant (*K_D_*) values of 6 to 7 μM (Table 1). Formation of the ternary Clr–cAMP–CBS-DNA complex is enthalpically driven with an opposed loss of entropy, whereas that of the binary Clr-cAMP complex relies both on enthalpic and entropic contributions. In contrast, cGMP binding by Clr alone proceeds endothermically and with apparently lower affinity (*K_D_*, ~24 μM). Compared to the Clr–cAMP–CBS-DNA complex, ternary complex formation with cGMP gives similar affinities and also relies on both contributions. For Clr alone, i.e., without cAMP or cGMP, no DNA binding could be detected under ITC conditions. In the apo state of E. coli CAP, the cAMP and CBS-DNA binding domains appear as quasi-independent dynamic units, while the intra- and intersubunit correlations increased in the holo state (45). Our ITC data indicate a profound difference only for the binary Clr-cNMP complexes, which may affect formation of ensembles capable of binding to CBS-DNA. According to size exclusion chromatography, apo-Clr forms a monomer-dimer equilibrium in solution that favors the monomeric state, with 86%. For comparison, the best studied Clr homolog, E. coli CAP, is able to bind both cAMP and cGMP but is only activated by cAMP for DNA binding (46). The lack of activation in response to cGMP has also been found for M. tuberculosis Rv3676 and has been assumed to be a generic trait of CRP-like proteins (43).

**TABLE 1:** ITC measurements for complex assembly^*a*^

Cell	Injectant	*K_D_* (μM)	*n*	Δ*H* (kcal/mol)	Δ*G* (kcal/mol)	–*T*Δ*S* (kcal/mol)
Clr	cAMP	5.8 ± 1.4	1.02 ± 0.016	−2.22 ± 0.05	−7.16 ± 0.12	−4.94 ± 0.22
Clr, DNA	cAMP	6.7 ± 2.7	0.57 ± 0.019	−11.5 ± 0.77	−7.08 ± 0.16	4.42 ± 0.36
DNA	Clr, cAMP	6.0 ± 4.7	0.39 ± 0.201	−11.0 ± 7.30	−7.13 ± 0.10	3.87 ± 1.37
Clr	cGMP	23.8 ± 14.5	1.49 ± 0.081	0.29 ± 0.02	−6.31 ± 0.05	−6.60 ± 0.07
Clr, DNA	cGMP	10.7 ± 3.9	0.57 ± 0.022	−4.91 ± 0.02	−6.79 ± 0.09	−1.88 ± 0.19
DNA	Clr, cGMP	4.5 ± 12.4	0.45 ± 0.408	−3.47 ± 5.33	−7.30 ± 0.02	−3.83 ± 0.02

a The measurements were conducted at 25°C. The titrant was injected stepwise from a syringe to the titrand solution (Clr, DNA or Clr-DNA) in the cell. ITC profiles were fit to a model with a single binding site (1:1); values for the equilibrium dissociation constant (KD), the enthalpy (ΔH) and the stoichiometry (n) were determined by curve fitting using Origin 7 software; values for free enthalpy (ΔG) and the entropic contribution (-TΔS) are therefrom calculated by the thermodynamic equations. Data are obtained from the average of three titrations. The corresponding raw data can be found in Fig. S3.

### Both cAMP and cGMP are able to shift Clr to its active conformation.

Unlike apo-Clr and its binary complexes, we found that ternary Clr–cNMP–CBS-DNA complexes crystallize within a few hours in the same, orthorhombic crystal form. Given their intrinsic high anisotropic diffraction characteristics, we employed extensive optimization screening to yield crystals diffracting to 2.8 and 3.1 Å for the cAMP- and cGMP-bound Clr–CBS-DNA complex, respectively (Protein Data Bank [PDB] accession codes 7PZA and 7PZB) (Table 2). The structures show a Clr dimer that is bound to CBS-DNA. The individual subunits fold into two domains linked by helix α5 (residues P123 to L149), which is the Clr dimerization interface. The α5 helices of the dimer form a coiled-coil mainly driven by hydrophobic interactions, with T140 and T141 as notable exceptions. Residues M1 to Q121 belong to the cNMP binding domain, the larger of the two. It is formed by four α helices and seven β strands in an antiparallel orientation of a β-sandwich encasing the cNMP binding site. An HTH DNA binding domain consisting of four α helices and two β strands is located at the C terminus (residues Y150 to D234). The cNMP binding domain is universally conserved throughout cNMP receptor proteins, as well as protein kinases and ion channels, with sequence identities above 20% even for mammal representatives (17). The HTH motif of the DNA binding domain is prevalent in 116 structures so far deposited in the PDB according to InterPro classifications (47, 48). Thus, both domains perform their function as core components of a variety of proteins adapted to fulfill different biological tasks. Accordingly, the overall fold of Clr, which belongs to the more diverse cluster G of the Crp/Fnr superfamily (Fig. S5), is highly similar to that of members of the main Crp cluster, namely, the catabolite activator protein (CAP) from E. coli (PDB accession code 1CGP) (49) and even more so to the M. tuberculosis (3MZH) and C. glutamicum (4CYD) CRPs (50, 51). Accordingly, the latter two served as the templates for molecular replacement.

**TABLE 2:** Data collection and refinement statistics for cNMP-bound Clr-DNA complex crystal structures^*a*^

Parameter	Value for:	
	Clr-cAMP-DNA complex (7PZA)	Clr-cGMP-DNA complex (7PZB)
Data collection statistics		
X-ray source	DESY P13	SLS PXI
Space group	P2_1_2_1_2_1_	P2_1_2_1_2_1_
Unit cell dimensions		
*a*, *b*, *c* (Å)	50.1, 69.9, 200.4	50.7, 72.9, 200.0
α = β = γ (°)	90	90
Wavelength (Å)	0.976	1.000
Resolution range (Å)	57.34–2.72 (2.82–2.72)	49.15–3.12 (3.23–3.12)
Completeness (%)	74.89 (6.72)	70.19 (5.53)
No. of observed reflections	137,679 (927)	41,837 (146)
No. of unique reflections	14,734 (129)	9,719 (74)
Multiplicity	9.3 (7.2)	4.3 (2.0)
Wilson B factor (Å^2^)	64.80	100.50
*R*_merge_ (%)	0.145 (1.223)	0.056 (0.481)
*R*_meas_ (%)	0.154 (1.315)	0.064 (0.621)
Mean *I/*σ(*I*)	11.35 (1.96)	11.08 (1.87)
CC_1/2_^*b*^	0.994 (0.788)	1.000 (0.610)
Refinement statistics		
Resolution range (Å)	57.34–2.72	49.15–3.12
*R*_work_*/R*_free_ (%)	0.271/0.303	0.289/0.333
Avg B factor (Å^2^)	68.16	96.83
No. of atoms		
Total	4,588	4,694
Protein/DNA	4,537	4,627
Ligand	46	46
Solvent	5	3
RMSD		
Bond length (Å)	0.021	0.005
Bond angle (°)	2.34	1.09
Ramachandran plot (%)		
Favored	88.81	88.20
Allowed	6.26	8.45
Outliers	4.92	3.34

a Values for the highest-resolution shell are given in parentheses.

b CC_1/2_ is the correlation coefficient between two random half data sets.

10.1128/mbio.03028-22.5 FIG S5 Differences for sequence motifs in the cNMP binding site and the hinge/α5 and α6 regions. Download FIG S5, PDF file, 0.2 MB. Copyright © 2023 Werel et al. 2023 Werel et al. https://creativecommons.org/licenses/by/4.0/ This content is distributed under the terms of the Creative Commons Attribution 4.0 International license.

**FIG 6:**
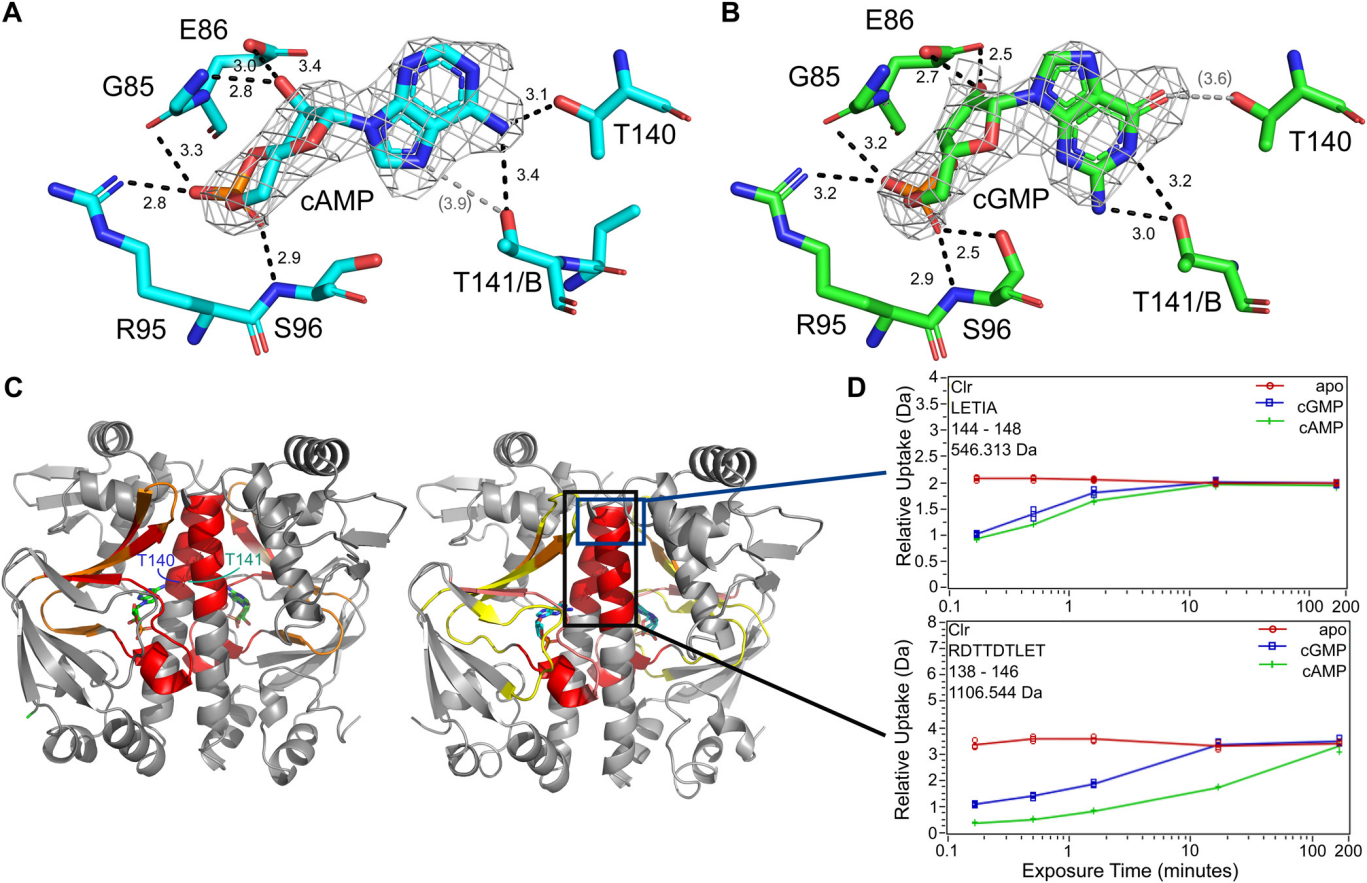
Characteristics of the Clr-ligand interaction. (A and B) Nucleotide binding environment in the cAMP- and cGMP-bound crystal structures. The sugar and phosphate moiety of the ligand interact with G85 and E86 of helix α4, as well as R95 and S96. The side chain of Ser96 is tilted toward the ligand in the cAMP-bound structure, and Thr141 of the adjacent subunit is in closer proximity to it. The primary amine of the adenosine is coordinating Thr140 from α5 in the same subunit and Thr141 from the adjacent one. The ligand 2 m*F*_o_ − D*F*_c_ experimental electron density maps contoured at 1.3 σ are shown in gray. Predicted hydrogen bonds (black dotted lines) are shown in angstroms; values in parentheses are too distant for indicating proper H-bonds. (C) Areas of reduced relative hydrogen-deuterium exchange upon binding of cAMP (left) and cGMP (right) colored from 5 to 20% in 5% increments (yellow, orange, red). In addition to the expected displacement of solvent in the binding cavity, the C-terminal half of helix 5 (black box) shows a reduction in exchange that can be attributed to a coil-to-helix transformation. (D) Time-dependent deuterium exchange graphs for two key peptides within helix 5 show that the shielding cannot be due solely to interaction with T140 and T141 but is the result of a change in secondary structure. Peptides not including both T140 and T141, exemplified by ^144^LETIA (upper box), also show a decrease in hydrogen/deuterium exchange.

The CBS-DNA interacts with its symmetry mate in the crystal lattice and is therefore well resolved. When the ternary complexes are compared, the Clr-cNMP structures as well as the CBS-DNA show a high conformational congruity with pairwise root mean square deviation (RMSD) values below 0.6 Å. This further supports our notion that both cyclic nucleotides shift ternary Clr–cNMP–CBS-DNA complexes to almost identical active conformations. The cNMP binding site located in the N-terminal domain is stoichiometrically occupied by cAMP and cGMP, respectively (Fig. 6A and B; Fig. S4). The binding modes of the phosphate and sugar moieties are identical, involving G85 and E86 within α4 as well as R95 and S96 of the following loop. The side chain of S96 is flipped toward the phosphate only in the cGMP-bound complex. The nucleobase of cGMP is flipped to the *syn* conformation, while it occupies the *anti* conformation in the Clr-cAMP complex. Both nucleobase conformations allow for interaction with helix α5 residue T140 of the same Clr subunit and T141 of the adjacent Clr subunit. While cAMP interacts via its primary N^6^-amine with both T140 and T141, cGMP forms hydrogen bonds between T140 and its C-6 carbonyl group as well as between T141 and the N^2^-amine. The latter interaction between the N^2^-amine of cGMP and T141 of the adjacent molecule is a substantial difference between Clr and the inactive cGMP complex of E. coli CAP (52). Interaction of the cAMP nucleotide with both α5 helices has been described to trigger a coil-to-helix transition in the C-terminal part of the hinge and to shift the HTH domain toward its active conformation for CAP (53). So far, cGMP appeared to be incapable of such an interaction with the second helix for promoting CRP activation (46). In the case of Clr, both the interaction and arrangement of the full α5 helix are resolved by the structure, which is in an agreement with Clr-DNA binding *in vitro* and activation of promoter activity *in vivo* by both cNMPs. Moreover, mapping the relative hydrogen-deuterium exchange between apo and holo states by HDX-MS experiments shows that upon nucleotide addition, the C-terminal half of the helix gets shielded from exchange. This further supports the notion that both nucleotides are capable of triggering the coil-to-helix transition in the linker to the C-terminal helix-turn-helix domain (Fig. 6C and D). This shielding is not due only to nucleotide interactions with T140 and T141, as even fragments, containing the last five residues of the α5 helix, show a clear decline in solvent accessibility for nucleotide-bound states. Helix α6 is shortened in response to the hinge rearrangement and interacts with the former at an angle of about 40° in the active conformation (11). This interaction is stabilized in Clr by a formerly unknown chloride-binding site. The relative hydrogen-deuterium exchange comparing the apo and holo states also shows a slightly stronger decline in deuterium uptake within the binding pocket for the cAMP-bound state than for the cGMP-bound state. Some uptake differences within helix α7 and α8 of the HTH domain can be observed (Fig. S4). Upon nucleotide binding, the deuterium exchange increases in this region, indicating some degree of conformational change. This agrees with structures published for E. coli CAP, where the active conformation is achieved by a 60° rotation of α8 (46, 54, 55).

10.1128/mbio.03028-22.4 FIG S4 Structural features of DNA binding by Clr. Download FIG S4, PDF file, 0.6 MB. Copyright © 2023 Werel et al. 2023 Werel et al. https://creativecommons.org/licenses/by/4.0/ This content is distributed under the terms of the Creative Commons Attribution 4.0 International license.

### The crystal structures of nucleotide-bound Clr shed light on its DNA specificity.

Clr binds to the target DNA via electrostatic interactions and hydrogen bonding to the major groove. The surface of the HTH DNA binding domain has a strongly positively charged patch with which it interacts with the DNA phosphate backbone (Fig. 5B). Clr has a likewise positively charged cavity in each subunit for accommodating cNMP. Helix α8 is inserted in the major groove and interacts with hydrogen bonds over six bases, as depicted in Fig. 5C and D. The binding results in a 40° and a 32° kinking of the DNA helix at the longer and shorter DNA oligomers, respectively, which is eased by the engineered strand breaks at the 2-fold symmetry center. The entropic cost associated with DNA bending is overcompensated by establishing further interactions. As a result, the flexibility of the flanking regions of the recognition sequence might be distinguished by Clr, adding another layer of specificity (56, 57).

The electron density of the duplex DNA that was annealed from four oligonucleotides corresponds to the 14 bp of the 5′ oligonucleotides of the core as well as 19 bp of the two 3′ oligonucleotides. The base readout occurs at most conserved positions of the core CBS motif (Fig. 5A) (24). Q184 of α7 and R195 and N199 of α8 are interacting with the ^9^TGT triplet of the 14-mers, whereas K197 and S194 of the same helix, as well as L152 within the loop next to the C-terminus of α5 are hydrogen bridged to the ^4^GGT triplet of the 19-mer. For E. coli CAP, mainly R180, E181, and R185 have been identified to interact with the recognition sequence (58, 59). In Clr, R180 corresponds to R195, E181 is not conserved, and R185 is conserved as R200 but fails to interact with the DNA as it adopts a different rotamer. Accordingly, proteins in the phylogenetic Crp main cluster, like E. coli CAP, have a highly conserved RE(T/M)VGR motif in their α8 helix, whereas members of cluster G, to which Clr belongs, are significantly more diverse in this region (16). Sequence differences between the main Crp cluster and the G cluster are not restricted to the DNA binding site but also occur in the motifs surrounding the cNMP-binding site, including the hinge/α5 region and the α6 helix. The higher sequence variation found for members of cluster G than for the Crp main cluster (Fig. S5) may correlate with the lower specificity for activation by cyclic nucleotides.

CRP proteins interact with the bacterial RNAP via the stacked β-strands within the HTH domain that has been termed AR1 in class I promoters (58, 60) or the top of the β-barrel within the cNMP binding domain, which has been labeled AR2, in class II promoters (61, 62). For Clr AR1, three of the four β-strands are present, whereas the N-terminal loop of the motif is turned more toward the protein core (Fig. S4). Crystal structures of CAP in complex with DNA and the carboxyl-terminal domain (αCTD) of the alpha-subunit of RNAP show very little flexibility within the interface and surprisingly only one hydrogen bond between T158 of CAP and T285 of the αCTD (PDB accession codes 5CIZ, 3N4M, and 1LB2). The interface is largely hydrophobic, a feature that is retained in Clr. The activating region AR1 has a significantly divergent orientation in its N-terminal part for Clr. This does prevent interaction with the E. coli RNAP *in vivo*, which we infer from promoter activation by Clr in E. coli (see above). The overall conservation of the 22 residues comprising AR1 is rather low, with three identical and three similar amino acids. In class II promoters, the RNAP interacts with the CRP on two additional sites: AR1 of the second dimer symmetry unit and AR2 of this unit (3). The interactions with AR2 involve the beta and omega subunits as shown in crystal and cryo-electron microscopy (cryo-EM) structures from Thermus thermophilus and E. coli (62, 63). The interactions of Clr within the omega subunit are conserved as G47 and D48 (G14 and E15 in T. thermophilus), whereas the area involved in the interaction with the beta subunit in T. thermophilus (mainly through E8) deviates in a way that allows no deductions on whether it would interact in the same way.

## DISCUSSION

Our study was focused on the dual specificity of Clr for cAMP and cGMP. We confirmed these binding specificities of Clr *in vitro* and characterized the binding properties of this transcription regulator for both these cNMPs and for its DNA binding site. In our ITC measurements, cAMP bound to Clr with about four times higher affinity than did cGMP (*K_D_*^cGMP^ = 24 μM; *K_D_*^cAMP^ = 6 μM). For CAP from E. coli, values in the same range have been reported, the affinity for cAMP (10 μM) also being greater than that for cGMP, at 16 μM (9, 42). However, we showed that the difference between the nucleotide affinities of Clr diminished as soon as cognate DNA was present, showing that ternary complex formation by both effector molecules is similarly driven.

The regulation of cAMP receptor proteins depends on an equilibrium between an active form that is capable of DNA interaction and an inactive form that is not. The equilibrium is shifted toward the active conformation by effector binding (18). For Clr, both our crystal structures and the HDX experiments clearly prove that Clr undergoes this switch independently of the purine base of cNMP. As the cNMP binding site does not deviate significantly from that of the E. coli homologue, Clr might have a lower energy barrier for the shift in population toward the active conformation. This is further supported by similar equilibrium shifts that have been introduced via mutations of E. coli CAP before (64). cAMP binding initiates the population shift by interacting with residues of the central helices of both subunits (CAP:T127 and CAP:S128) (65). This in turn leads to a coil-to-helix transition in the C-terminal part of the helix, resulting in a DNA binding domain in its active conformation (11, 54, 66, 67). In contrast to any previous findings in E. coli, we clearly established that Clr performs an identical binding movement via a ^140^TT motif for both cNMPs. As cGMP is bound in a *syn* conformation, the interactions can be retained. We were able to show the resulting coil-to-helix switch of the α5 helix by HDX-MS in solution due to a decline in deuterium uptake, further proving the dual cNMP specificity nature of Clr. Clr residues G85, E86, R95, and S96, which are also part of the binding pocket, are largely conserved in CAP as well and responsible for the high-affinity nucleotide binding (68).

The C-terminal HTH motif of CRPs recognizes its cognate consensus DNA motif via major groove readout (45). Binding of the Clr dimer to two symmetrically related motifs results in a kink of the DNA by 40° and 32°. For CAP, DNA kinks of even 110° have been reported and modified promoters containing an inherent kink have been shown to function independently of CRP, which highlights the importance of this particular feature (49, 59, 69–71). Our ITC measurements of DNA binding gave dissociation constants of 6.0 and 4.5 μM and free energy changes of −7.1 and −7.3 kcal/mol for cAMP and cGMP, respectively. This makes Clr a transcription factor on the weaker side of the binding spectrum. However, stronger does not always equal better, as the binding needs to be reversible as well (56). For duplex DNA of the similar-length CAP dimer·cAMP_2_, binding affinities of about 10 μM have been reported, which suggests similar binding strengths for CAP and Clr (71).

Clr interacts with the nucleobases of the DNA core motif via hydrogen bonds. The residues involved in sequence readout are exactly contacting the bases within the CBSs as derived from genome-wide ChIP-seq and EMSA data of this work. The dual specificity of Clr for cAMP and cGMP was demonstrated *in vivo* as well. Virtually all promoters directly targeted by Clr were activated by Clr-cAMP and Clr-cGMP in both S. meliloti and E. coli. The arrangement of three overlapping imperfect CBS core motifs in the *SMc05008* upstream region is intriguing. It suggests a binding mode different from the binding of a single CBS or that differential binding of the individual sites might generate a different regulatory output. In S. meliloti, we found that externally added cGMP activated gene expression up to four times higher than did cAMP. Both the affinities and structures of cNMP-bound Clr showed no clear distinction, explaining the higher degree of cGMP-dependent promoter activation in S. meliloti. Moreover, stronger cGMP-mediated activation was not observed in E. coli. The difference is therefore likely rooted in higher cGMP levels within the S. meliloti cell under the *in vivo* assay conditions used in this study. Whether this is caused by endogenous AC/GCs, differences in uptake of exogenous cNMP, or different cAMP and cGMP degradation rates remains to be elucidated. Previously, intracellular cGMP was detected in S. meliloti wild-type cultures, but levels of cGMP were 20-fold lower than levels of cAMP (5). This cGMP may originate from side activities of enzymes with primary AC activity, since no dominant GC activity has yet been found for any of the putative AC/GC enzymes in S. meliloti. Small amounts of cGMP were detected in the culture supernatant of an S. meliloti CyaB overproduction strain (24). Which cNMP molecules are synthesized under native conditions and at what levels are not yet known, as concentrations seem to be rather low.

Except for *cyaF2*, which encodes a putative AC or GC, all other identified genes directly regulated by Clr-cNMP code for hypothetical proteins. Eight of the Clr-cNMP-regulated genes encode small proteins of up to 100 amino acids, six of these with an N-terminal signal peptide. *SMb20495*, *SMc02177*, and *SMc02178* predicted to encode hypothetical proteins were previously shown to participate in autoregulation of alfalfa root hair infections (5, 31, 72). With the exception of these three genes, the biological role of the other identified Clr-cNMP targets remains unclear. Because many of the directly Clr-cNMP controlled target genes encode proteins with an N-terminal signal peptide, we speculate that most of these proteins might be relevant for extracellular functions and possibly involved in the symbiotic interaction with the host plant.

Notably, Clr-cNMP was found to directly induce transcription of the small noncoding RNA *SMelC181* of unknown function. Only a few studies reported regulation of small noncoding RNAs by a CRP. Examples are small noncoding RNAs associated with the regulation of quorum sensing, nitrogen assimilation, and galactose utilization in E. coli, Salmonella, *Vibrionales*, and cyanobacteria (73–75). This makes *SMelC181* an interesting candidate for upcoming studies.

In the last 2 decades, cNMP signaling networks have been characterized in several bacterial species and different CRPs have been studied for their cNMP and DNA binding properties (3, 6, 10, 76). These studies imply that cNMP signaling networks and CRPs have evolved differently in the context of the different habitats of these bacteria. In this study, we showcased a CRP-like protein that has evolved to be activated both by cAMP and cGMP to perform its function in transcriptional regulation in a plant-symbiotic alphaproteobacterium. Clr is well conserved in the *Sinorhizobium* genus, including the cAMP-binding and the unusual DNA-binding amino acid motifs. In other closely related symbiotic nitrogen-fixing rhizobia, such as Rhizobium leguminosarum and Rhizobium etli, Clr homologs have not been found. However, similar proteins are present in individual *Rhizobium* species and Bradyrhizobium diazoefficiens isolates. The biological function of Clr dual specificity for activation by the two cNMPs remains to be discovered. It might have evolved as an adaptation to endogenous bacterial cGMP synthesis. However, conditions leading to decent levels of cGMP in S. meliloti are unknown. A role for cGMP in plant signal transductions related to development, stress response, ion homeostasis, and hormone function has been demonstrated, and long-distance cGMP signaling was suggested (77, 78). It is therefore tempting to speculate that S. meliloti may sense host plant-derived cGMP. Although the S. meliloti Clr regulon has been extensively characterized, current knowledge of its biological functions is limited to its role in early stages of the symbiosis (5). Deciphering the biological function of the responsiveness of Clr to cAMP and cGMP and whether cluster G CRP transcription factors are generally characterized by being activatable by cGMP or by both cGMP and cAMP is an exciting goal of future research.

## MATERIALS AND METHODS

### Strains, plasmids, and growth conditions.

Unless specified otherwise, E. coli was grown at 37°C in LB-Lennox medium (1% tryptone, 0.5% yeast extract, 0.5% NaCl) with the addition of kanamycin (50 μg/mL), gentamicin (8 μg/mL), or tetracycline (10 μg/mL) as needed. S. meliloti was grown in TY medium (0.5% tryptone, 0.3% yeast extract, 2.7 mM CaCl_2_) or MOPS minimal medium (1% MOPS, 1 mM MgSO_4_, 20 mM sodium glutamate, 20 mM mannitol, 2 mM K_2_HPO_4_, 250 μM CaCl_2_, 37 μM FeCl_3_, 4.1 μM biotin, 45.8 μM H_3_BO_3_, 10 μM MnSO_4_, 1 μM ZnSO_4_, 0.5 μM CuSO_4_, 0.27 μM CoCl_2_, 0.5 μM Na_2_MoO_4_). If required, streptomycin (600 μg/mL), kanamycin (200 μg/mL), gentamicin (30 μg/mL), or tetracycline (10 μg/mL) was added to solid medium containing agar as a solidifying agent. For liquid media, the concentrations of kanamycin, gentamicin, or tetracycline were reduced by half.

Plasmids were transferred to S. meliloti by E. coli S17-1-mediated conjugation as previously described (79).

Promoter-probe constructs were generated by insertion of an S. meliloti gene upstream region of 300 to 735 bp and up to 75 bp of the associated protein or RNA coding region into the replicative low-copy-number plasmid pPHU231-EGFP or replicative medium-copy-number plasmid pSRKKm-EGFP for studies in S. meliloti strains. For the promoters of small noncoding RNA genes, a Shine-Dalgarno sequence was included downstream of the selected promoter sequence.

Site-directed mutagenesis of putative CBSs was performed by overlap extension PCR, and mutated promoter regions of the same size as the corresponding wild-type promoter region were inserted into pSRKKm-EGFP.

For cloning of CBSs, two complementary oligonucleotides (1 μM each) were hybridized in T4 DNA ligase buffer (NEB) containing T4 polynucleotide kinase (PNK; 0.2 μL) (NEB) in a total volume of 10 μL to obtain double-stranded DNA flanked by XbaI and HindIII overhangs. Phosphorylation of the oligonucleotides was performed at 37°C for 45 min, followed by a PNK deactivating step at 65°C for 20 min. The reaction mixture was then heated at 95°C for 5 min and slowly cooled down to 30°C. Thirty nanograms of the hybridized product was directly used as an insert for ligation with pSRKKm-EGFP.

For construction of the C-terminal 3×FLAG-tagged Clr overexpression construct, the full-length *clr* coding sequence was amplified from genomic DNA of S. meliloti Rm2011, digested with NdeI and XbaI, and inserted into plasmid pSRKKm-CF, yielding pSRKKm-*clr*-CF. Subsequently, *clr*-CF was amplified from this plasmid, digested with XbaI and HindIII, and inserted into plasmid pWBT, yielding pWBT-*clr*-CF. Expression plasmids pWBT-AC/GC and pWBT-AC/GC-c*lr* are described in reference 24.

To obtain S. meliloti gene deletion mutants, gene-flanking regions of 350 to 750 bp were cloned into suicide vector pK18mobSacB. Following conjugation-mediated plasmid transfer to S. meliloti Rm2011 and plasmid integration into the genomic DNA by homologous recombination, transconjugants were subjected to sucrose selection as previously described (80). Gene deletions were verified by PCR. The S. meliloti *cya^0^* multiple deletion mutant lacking the annotated 28 putative class III AC/GC genes was generated by sequential gene deletions. The genomes of *cya^0^* strains as well as the parenting strain Rm2011 were sequenced. Total DNA was purified using a DNeasy blood and tissue kit (Qiagen). DNA sequencing libraries were generated by applying the Nextera XT DNA library preparation kit (Illumina). Sequencing was performed on a MiSeq desktop sequencer (Illumina), using the MiSeq reagent kit v2 for 2 × 250-bp paired-end reads (Illumina) for Rm2011, resulting in 4.45 × 10^6^ reads, or the MiSeq reagent kit v3 for 2 × 75-bp paired-end reads for Rm2011 *cya^0^*, resulting in 5.82 × 10^6^ reads. Single-nucleotide polymorphism (SNP) detection was performed by applying CLC Genomics Workbench (v10.1.1; Qiagen). Paired reads were mapped to the annotated reference genome of S. meliloti Rm1021 (chromosome, GenBank accession no. AL591688; pSymA, NC_003037; pSymB, AL591985). The minimum variation frequency was 50%, and at least 10 reads with a minimum mismatch coverage of 8 were considered. The S. meliloti *cya^0^* strain used in this study (Fig. 4; see Table S3 in the supplemental material) carries one SNP (Table S1K).

To construct the expression plasmid coding for C-terminally His_6_-tagged Clr, the native encoding sequence was amplified by PCR and inserted into NdeI- and XhoI-digested pET36b(+) (Novagen). The fusion construct was verified by DNA sequencing.

Strains, plasmids, and oligonucleotides used in this study are described in Table S5A and B.

10.1128/mbio.03028-22.10 TABLE S5 Strains and plasmids, EMSA DNA fragments, mutated DNA fragments, and oligonucleotides. Download Table S5, XLSX file, 0.05 MB. Copyright © 2023 Werel et al. 2023 Werel et al. https://creativecommons.org/licenses/by/4.0/ This content is distributed under the terms of the Creative Commons Attribution 4.0 International license.

### EGFP fluorescence measurements.

S. meliloti strains were grown in 100 μL TY medium, and E. coli BTH101 was grown in 100 μL LB medium in 96-well polystyrene flat-bottom plates (Greiner) at 30°C with shaking at 1,200 rpm. For cultures in which cNMPs were added to the medium, stationary precultures of the respective strains grown in TY medium without cNMPs were diluted 1:100 in medium containing 400 μM cAMP or cGMP or as a control in medium without cNMPs, followed by incubation for 24 h. For promoter studies in E. coli BTH101, stationary precultures were diluted 1:100 in medium containing 100 μM IPTG, followed by incubation for 16 h.

EGFP fluorescence measurements were carried out as described previously (24). Relative fluorescent units (RFU) represent fluorescent values divided by the optical density at 600 nm (OD_600_). The measurements were carried out in S. meliloti Rm2011, Rm2011 Δ*clr*, and Rm2011 *cya^0^* and in E. coli BTH101 strains. Processed data and underlying raw data of each experiment are listed in Tables S2 to S13.

In S. meliloti strains, the background fluorescence of the corresponding empty vector control was subtracted (Fig. 1A and C; Fig. S2E and Tables S2 and S3). In measurements carried out in E. coli BTH101 (Fig. 4B; Table S4), no background fluorescence was subtracted. Fluorescence fold change of S. meliloti strains carrying promoter-probe constructs was calculated from the RFU of the induced strains divided by the RFU of the noninduced strains or by the RFU of the wild type divided by the RFU of the mutants (Table S2). In E. coli, fluorescence fold change was calculated using the fluorescent values derived from the strains carrying pPHU231-promoter-probe-EGFP in combination with pWBT-AC/GC-*clr*-EGFP divided by the values derived from the strains carrying pPHU231-promoter-probe-EGFP in combination with pWBT-AC/GC-EGFP (Table S4). Three to four independent transconjugants and transformants of each strain containing the promoter-*egfp* constructs were used as biological replicates.

### ChIP-seq.

Cultures of Rm2011 Δ*clr* carrying the *clr*-FLAG overexpression construct pWBT-clr-CF were grown in 60 mL of TY or MOPS medium in 500-mL flasks, each supplemented with either 400 μM 3′,5′-cAMP or 3′,5′-cGMP and 500 μM IPTG. Upon reaching an OD_600_ of 0.6, the cells were fixed with 1% formaldehyde for 20 min at room temperature. Fixation was quenched with 250 μM glycine for 20 min.

Cell pellets were resuspended in 300 μL immunoprecipitation (IP) buffer (50 mM HEPES-KOH [pH 7.8], 150 mM NaCl, 1 mM EDTA, 1% Triton X-100, 0.1% sodium deoxycholate, 0.1% SDS) with 1 mM phenylmethylsulfonyl fluoride (PMSF). Cells were lysed and DNA fragmented (~500 bp) by sonication in a Bioruptor Plus (Diagenode) by 48 cycles of 30 s of sonication and 30 s of cooling. Three hundred microliters of sonicated product was mixed with 3 mL of IP buffer and 1 mM PMSF and centrifuged at full speed at 4°C for 30 min. One hundred microliters of supernatant was taken and frozen at −20°C as a control. Clr-CF was immunoprecipitated with Anti-FLAG M2 affinity gel (Sigma-Aldrich). The protein was eluted with 100 μL of 3×FLAG peptide elution buffer for 1 h at 4°C. Cross-links of control and ChIP DNA were removed with 200 μM NaCl, and proteins and RNA were degraded with proteinase K and RNase A overnight at 65°C. Samples were checked by Western blotting with monoclonal anti-FLAG M2-horseradish peroxidase (HRP) antibody (1:1,000). DNA was purified with the QIAquick PCR kit. Ten nanograms of DNA was taken for ChIP-seq DNA library preparation and 2.5 ng for quantitative PCR (qPCR). The DNA library preparation was carried out as described previously (81) with the adapter sequences 2, 4 to 7, and 12 to 14 compatible with Illumina’s TruSeq platform. Sequencing was performed on a MiSeq desktop sequencer (Illumina) using a MiSeq reagent kit v3 with 2 × 75 paired-end reads, and sequence analysis was performed using CLC Genomics Workbench (v10.1.1; Qiagen).

ChIP-seq reads 1 and 2 were paired with a distance between 1 and 1,000 bp, and failed reads were removed. Paired reads were mapped to the annotated S. meliloti 1021 genome (Chr1021 embl with annotation AL591688, pSymA1021 embl with annotation AE006469, and pSymB1021 embl with annotation AL591985). The following mapping parameters were used: match score = 1, mismatch cost = 2, linear gap cost = 3 (deletion/insertion), length fraction = 0.5, similarity fraction = 0.8 (at least 50% of the alignment has to have 80% sequence identity). Nonspecific matches were mapped randomly but also assigned as such. Peak calling was performed with the transcription factor ChIP-seq tool by comparing the mapped reads of IP and control samples (maximum *P* value = 0.05). Sequences flanking each center of a peak derived from the ChIP-seq analysis were defined and summarized as FASTA sequences using the R package (82). Motifs were generated with the MEME Suite online tools MEME (v5.1) and FIMO (v5.3) (30, 83).

### EMSA.

An EMSA reaction mixture contained 2 mM HEPES (pH 7.0), 30 mM NaCl, 50 mM KCl, 850 ng of sonicated salmon sperm DNA (GE Healthcare), 1 μg of bovine serum albumin (BSA) (Sigma), and 20 ng of Cy3-labeled DNA in a final volume of 10 μL. Native and synthetic Cy3-labeled DNA fragments (Table S5C and D) were obtained by PCR with Cy3-labeled primers (Table S5B) using the corresponding pSRKKm-promoter-EGFP and pSRKKm-CBS-EGFP constructs as the template, respectively. Synthetic Cy3-labeled DNA fragments of 592 bp were composed of the CBS flanked by pSRKKm-EGFP-derived sequence. The protein was added at 3 μg (111 μM) per reaction mixture, and 3′,5′-cAMP or 3′,5′-cGMP was added at 1 mM if indicated. The reaction mixtures were incubated at room temperature for 30 min in the dark. A mixture of 1 μL of 90% glycerol and 1.5 μL of 5× Tris-borate-EDTA (TBE) buffer was added to each reaction mixture, and 10 μL of this mixture was loaded onto a 10% polyacrylamide gel in 1× TBE. Following electrophoresis at 9 V cm^−1^ at room temperature for 3.5 h, images were taken using a Typhoon 8600 variable mode imager (Amersham Bioscience).

### Protein expression and purification.

Clr was overproduced in E. coli BL21(DE3) cells in LB-Lennox medium containing kanamycin (30 μg/mL) at 37°C and induced at an OD_600_ of 0.5 using IPTG (0.1 mM). Three hours after addition of 0.1 mM IPTG, Clr was harvested by centrifugation (15 min at 4°C; 4,000 rpm). The pellet was resuspended in 20 mL of binding buffer (50 mM sodium dihydrogen phosphate, 1,000 mM sodium chloride, 10 mM imidazole, pH 7.0) and disrupted by three passages through a cold French pressure cell press at 15.2 × 10^5^ Pa cm^−2^. The lysate was centrifuged at 18,000 rpm for 60 min at 4°C and filtered through 0.45-μm membrane filter. For Clr purification, the initial purification was conducted using a 5-mL Protino Ni-nitrilotriacetic acid (Ni-NTA) column (Macherey-Nagel). Size-exclusion chromatography over a 16/600 Superdex 200 column (GE) using elution buffer (20 mM HEPES, 300 mM NaCl, pH 7.0) was done as a polishing step. Preceding the purification, the size exclusion buffer was optimized as described using SYPRO orange dye (84).

### Protein crystallization.

Clr at 0.25 mM or 0.44 mM was crystallized in the presence of 25 mM cAMP or cGMP, respectively, 25 mM MgCl_2_, and a 1.25-fold excess of 19-mer/14-mer duplex target DNA (19-mer sense strand, 5′-CTA GGT AAC ATT ACT CGC G-3′; 14-mer antisense strand, 5′-GCG AGT AAT GTT AC-3′). The oligonucleotide (BioCat) was prepared from single strands by heating equimolar amounts to 98°C for 5 min and slowly cooling to room temperature. The DNA oligonucleotides used for the crystallization of Clr were designed based on the genes *SMc04190* and *SMc00925*, which have previously been reported to be regulated by Clr (24).

Crystals of Clr in complex with cAMP were grown at 291 K by sitting-drop vapor diffusion in 0.09 M sodium fluoride, 0.09 M sodium bromide, 0.09 M sodium iodide, 0.1 M Tris base/bicine (pH 8.5), 12.5% (vol/vol) 2-methyl-2,4-pentanediol (MPD), 12.5% (wt/vol) polyethylene glycol (PEG) 1000, and 12.5% (wt/vol) PEG 3350. Crystals containing cGMP were grown at 281 K in sitting-drop vapor diffusion plates in 0.2 M 1,6-hexanediol, 0.2 M 1-butanol, 0.2 M (*RS*)-1,2-butanediol, 0.2 M 2-propanol, 0.2 M 1,4-butanediol, 0.2 M 1,3-propanediol, 0.1 M MOPS/HEPES-Na (pH 7.5), 12.5% (vol/vol) MPD, 12.5% (wt/vol) PEG 1000, and 12.5% (wt/vol) PEG 3350.

The crystals were flash-frozen in liquid nitrogen, and diffraction data were collected at 100 K at the EMBL/DESY P13 beamline (Hamburg) using a Pilatus 6M detector (Dectris) and at the SLS PXI X06SA beamline (Paul Scherrer Institute, Villigen, Switzerland) using an Eiger 16M detector (Dectris), respectively. At wavelengths of 0.976 and 1.000 Å, the crystals diffracted to 2.8 and 3.1 Å, respectively. The data were processed using XDS (85) in space group P2_1_ 2_1_ 2_1_. Data reduction and scaling were done using CCP4i2 (v7.0.065) (86), AIMLESS in particular (version 0.7.3). The phases were solved by molecular replacement using a hybrid model derived from GlxR from Corynebacterium glutamicum (PDB code 4CYD) and Crp from Mycobacterium tuberculosis (PDB code 3MZH). The model was built using COOT (v0.8.9) (87) with refinement in Phenix (v1.11.1) (88). Final refinement statistics are given in Table 2. The coordinates and structure factors were deposited in the Protein Data Bank under PDB codes 7PZA and 7PZB.

### ITC measurements.

Clr was transferred to the binding buffer (20 mM HEPES, 300 mM NaCl, 20 mM MgCl_2_, pH 7.0) via a PD-10 column (Cytiva Life Sciences), and the titration calorimetry measurements were performed using a Malvern MicroCAL ITC calorimeter as previously described (89). A typical titration consisted of injecting 2-μL aliquots of 5 mM ligand solution into 0.3 to 0.4 mM protein solution every 2.5 min to ensure that the titration peak returned to the baseline prior to the next injection. For the measurement of DNA affinities, the setup was reversed, with protein titration at 0.3 to 0.4 M containing 1 mM cyclic nucleotide into DNA at 25 μM. The cell was temperature controlled to 25°C. Titration curves for the dilution of the ligand solution were deducted from the data.

### HDX-MS measurements.

Sample preparation was automated with a two-arm robotic autosampler (LEAP Technologies). A 7.5-μL volume of 50 μM Clr with and without 1 mM cNMP was mixed with 67.5 μL of D_2_O-based size exclusion chromatography (SEC) buffer. After 10, 30, 95, 1,000, and 10,000 s at 25°C, respectively, the hydrogen/deuterium exchange was quenched by the addition of equal parts quench buffer (400 mM KH_2_PO_4_/H_3_PO_4_, 2 M guanidine hydrochloride, pH 2.2) at 1°C. The solution was injected into an Acquity ultraperformance liquid chromatography (UPLC) M-class system with HDX technology (Waters). The protein was digested online with immobilized porcine pepsin at 12°C at 100 μL/min (H_2_O, 0.1% formic acid), and the resulting peptides were collected on a trap column (2 mm by 2 cm) with POROS 20 R2 material (Thermo Scientific) at 0.5°C. After 3 min, the trap column was switched online with an Acquity UPLC BEH C_18_ column (1.7 μm, 1.0 by 100 mm) (Waters), and the peptides were eluted at 0.5°C using a gradient of H_2_O–0.1% formic acid (A) and acetonitrile–0.1% formic acid (B) at 30 μL/min (5% to 35% B in 7 min, 35% to 85% B within 1 min, and isocratic flow 85% B for 2 min). The column was washed for 1 min at 95% B and equilibrated at 5% B for 5 min after this. Peptides were ionized by electrospray ionization at a 250°C source capillary temperature and a spray voltage of 3.0 kV. Mass spectra were acquired on a G2-Si high-definition mass spectrometer (HDMS) with ion mobility separation (Waters) over a range of 50 to 2,000 *m/z* in HDMSE (high-definition MS^E^) or HDMS mode for undeuterated and deuterated samples, respectively. Lock mass correction was performed with a [Glu1]-fibrinopeptide B standard (Waters). Between samples, the pepsin column was washed three times with 80 μL of 4% (vol/vol) acetonitrile and 0.5 M guanidine hydrochloride, and additionally, blank runs were performed between samples. Peptides were identified, and deuterium uptake was determined by employing the PLGS and DynamX 3.0 software suites (both from Waters) as described previously (90).

### Data availability.

The ChIP-seq data have been deposited in the ArrayExpress database at EMBL-EBI (https://www.ebi.ac.uk/arrayexpress) under accession no. E-MTAB-11788 (91). TSS data of S. meliloti strain RFF625c are available from ArrayExpress (E-MTAB-12127). The Clr protein crystal structure data have been deposited in the Protein Data Bank archive under accession codes 7PZA and 7PZB. All study data are included in the article and/or supplemental material.
