# Supplementary material for: Structural Basis of Dual Specificity of Sinorhizobium meliloti Clr, a cAMP and cGMP Receptor Protein
Source: mBio. 2023 Apr 5;14(2):e03028-22. doi: 10.1128/mbio.03028-22 (PMC10127613; doi:10.1128/mbio.03028-22)
Supplement: FIG S1 [file mbio.03028-22-s0001.pdf]

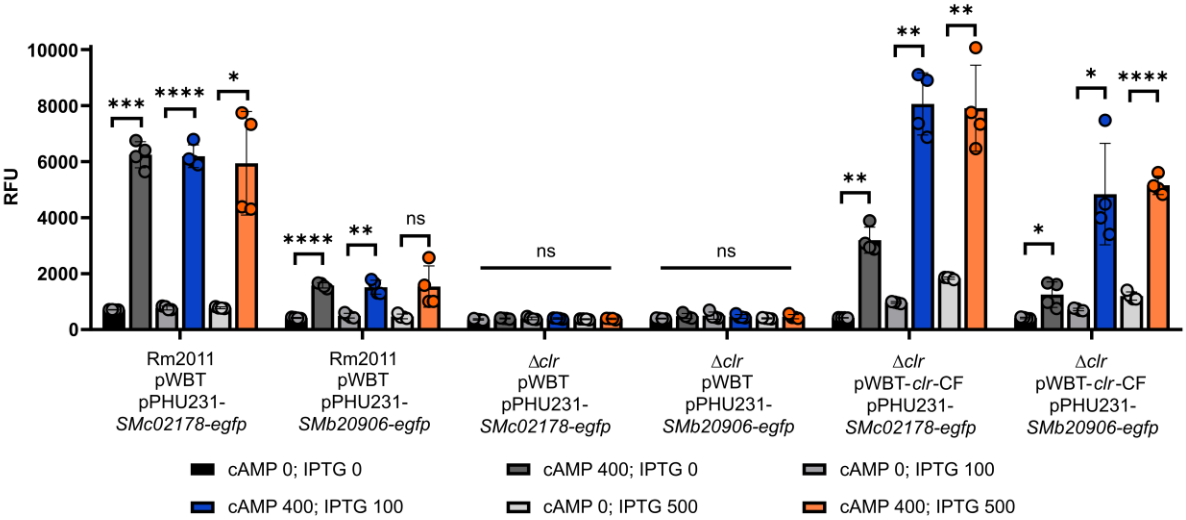

FIG S1 Functionality of Clr-CF. Relative fluorescent units (RFU) derived from measurements of promoter-*egfp* construct-mediated fluorescence upon IPTG-mediated induction of production of C-terminally FLAG-tagged Clr (Clr-CF). Measurements were carried out in wild type Rm2011 and Δ*clr* 24 h post induction with IPTG and cAMP at the indicated concentrations (µM). Bar plots show average RFU and their standard deviation derived of four biological replicates. Independent measurements are indicated as dots. Significance was calculated using student's t-test and p-values are indicated with asterisks (ns, $p > 0.05$; *, $p \leq 0.05$; **, $p \leq 0.01$; ***, $p \leq 0.001$; ****, $p \leq 0.0001$).
