# Supplementary material for: Structural Basis of Dual Specificity of Sinorhizobium meliloti Clr, a cAMP and cGMP Receptor Protein
Source: mBio. 2023 Apr 5;14(2):e03028-22. doi: 10.1128/mbio.03028-22 (PMC10127613; doi:10.1128/mbio.03028-22)
Supplement: FIG S2 [file mbio.03028-22-s0002.pdf]

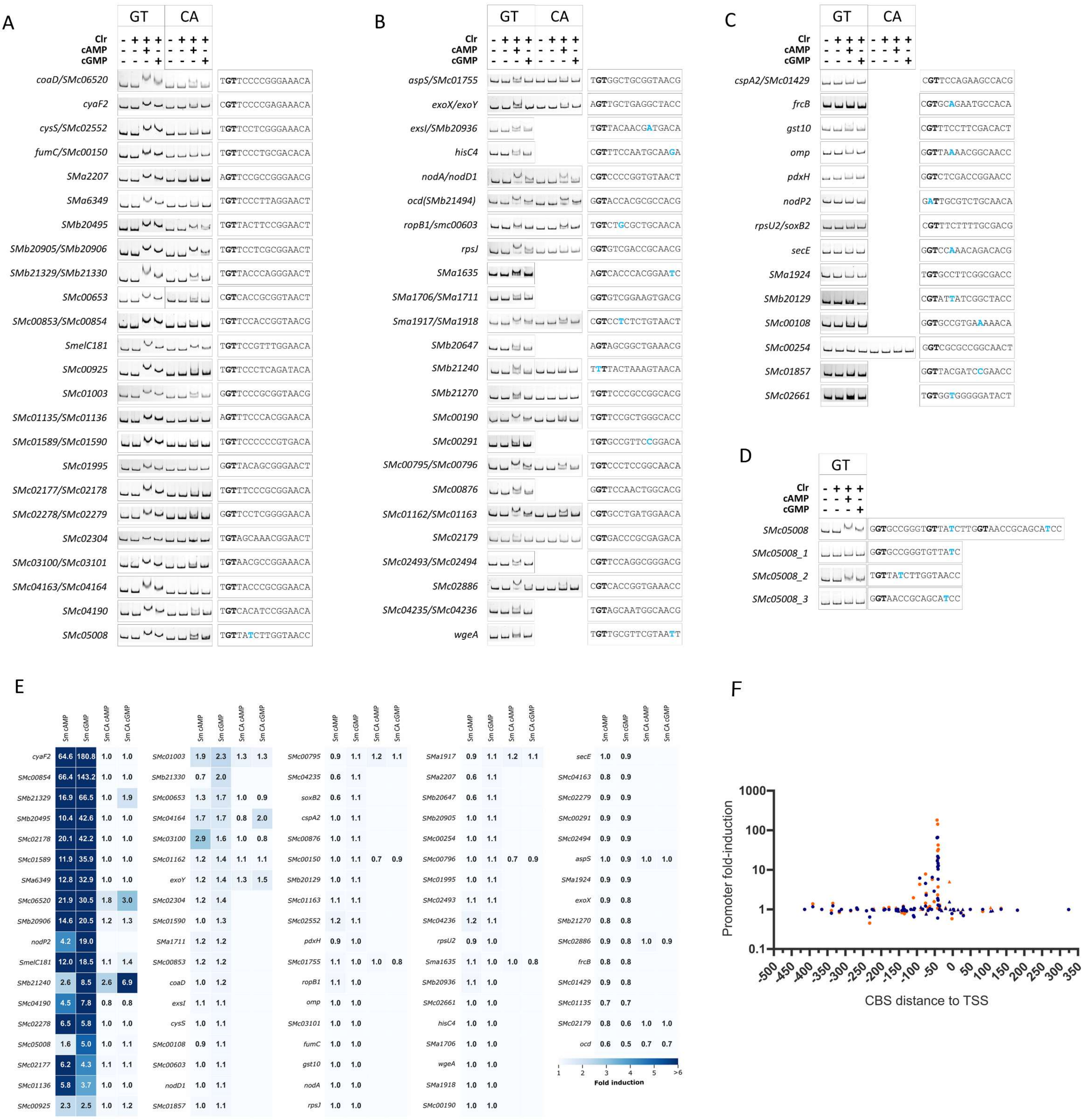

FIG S2 EMSA shift patterns of all putative Clr binding sites and promoter-probe measurements in *S. meliloti*. EMSAs are shown for all tested putative binding sites (right panel) with the native (GT) or mutated (CA) binding motifs. Binding sites are grouped by strong (A) weak (B) or no (C) binding. D) EMSAs for the native *SMc05008* promoter and the three independent binding sites of the *SMc05008* promoter. Mismatches are indicated in blue. (E) EGFP expression measurements were carried out in *S. meliloti* Rm2011 with three to four independent transconjugants as biological replicates. The heatmap displays the fold-change of EGFP fluorescent signal mediated by promoter-probe constructs carrying either the native (Sm) or mutated putative CBS (Sm CA) in cells cultured in medium supplemented with either cAMP or cGMP. Ratios derive from RFU of cNMP-induced against uninduced cultures. No data are available for empty squares. Values of each biological replicate measured, and standard deviation are given in Table S2. (F) Promoter-probe induction relative to CBS location. All promoter regions with TSS data available and containing a putative CBS with a perfect (circle) or imperfect (one mismatch) (triangle) consensus sequence are displayed. TSS were derived from RNA-seq data of *S. meliloti* wild type strains (33, 34) and of the *ecf*$^{-}$ *S. meliloti* strain RFF625c (91). Promoter induction ratios derived from *S. meliloti* cultures supplemented with cAMP (blue) or cGMP (orange) (this Figure panel E and Table S2).
