# Supplementary material for: Structural Basis of Dual Specificity of Sinorhizobium meliloti Clr, a cAMP and cGMP Receptor Protein
Source: mBio. 2023 Apr 5;14(2):e03028-22. doi: 10.1128/mbio.03028-22 (PMC10127613; doi:10.1128/mbio.03028-22)
Supplement: FIG S3 [file mbio.03028-22-s0003.pdf]

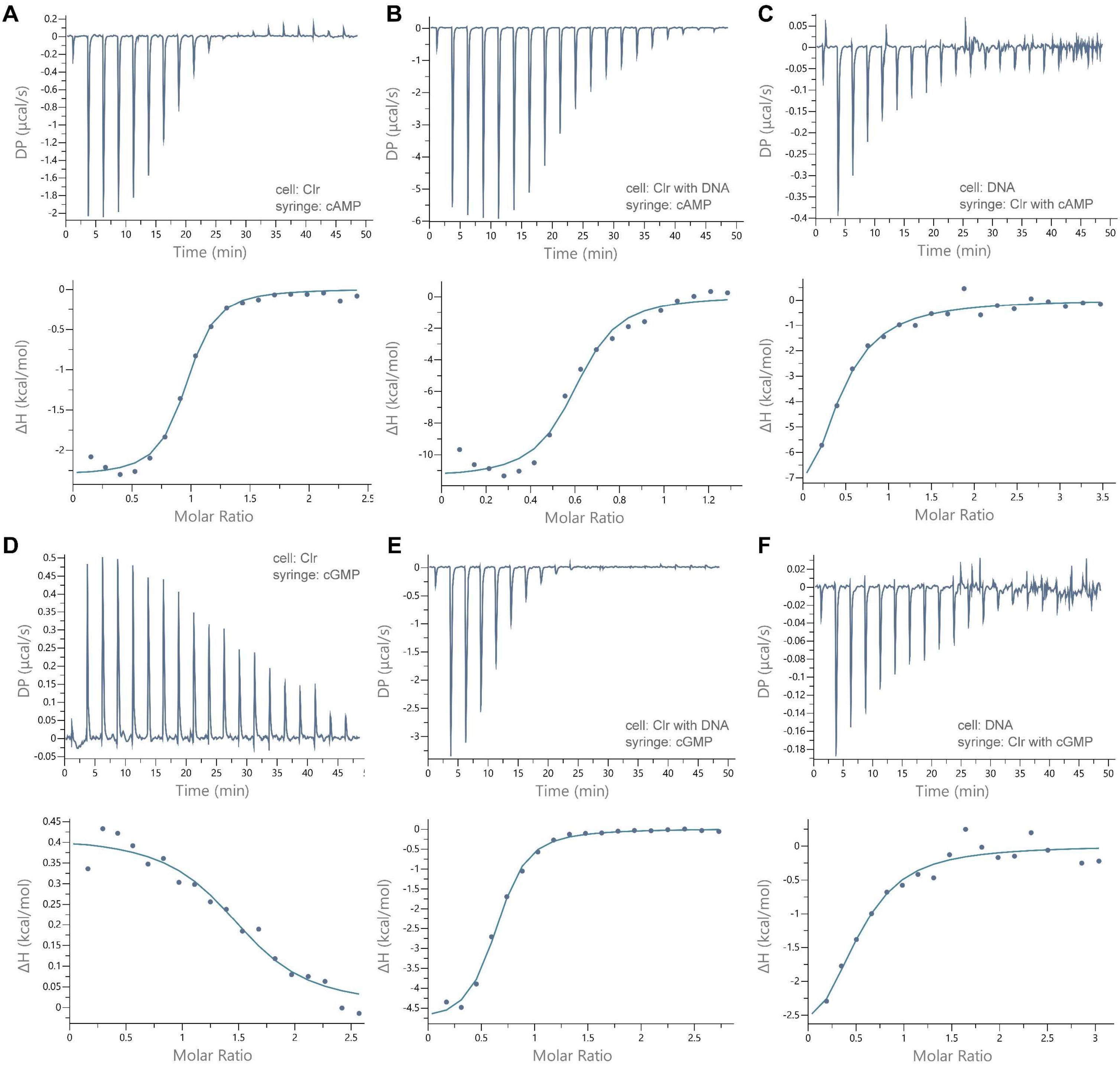

FIG S3 Raw and integrated ITC thermograms. (A, D) The interaction of Clr with cyclic adenosine monophosphate is exothermic, whereas cyclic guanosine binding is endothermic and has a lower affinity. (B) No major differences in binding behavior compared to (A) can be observed upon addition of cAMP to a sample containing the DNA oligo as well as Clr. (E) In contrast, the presence of DNA for cGMP shifts the binding to an exothermic behavior with increased affinity. (C, F) DNA binding facilitated by either cNMP is within the same range.
