# Supplementary material for: Structural Basis of Dual Specificity of Sinorhizobium meliloti Clr, a cAMP and cGMP Receptor Protein
Source: mBio. 2023 Apr 5;14(2):e03028-22. doi: 10.1128/mbio.03028-22 (PMC10127613; doi:10.1128/mbio.03028-22)
Supplement: FIG S5 [file mbio.03028-22-s0005.pdf]

CRP cluster

			β4			hinge			α6				
E. coli	P0ACJ8	*CAP*	D^{54}	S^{63}	R^{83}	R^{124}	T^{128}	S^{129}	D^{139}	G^{142}	R^{143}	A^{145}	L^{149}
			S^{a},H^{b}	F^{b}	G^{a}	N^{c}	L,C,I^{b}	T^{b}	N,K^{b}	S,Q,K,D^{b}	C^{b}	T,S,Q,Y,L,F,V,C^{b}	K^{a}, R^{b}
P. aeruginosa	P55222	*Vfr*	D	S	R	D	T	S	D	G	R	A	L
M. tuberculosis	P9WMH3	*Crp*	D	S	R	R	T	S	D	G	R	A	L
Y. pestis	Q79RU4	*Crp*	D	S	R	N	T	S	D	G	R	A	L
V. cholerae	D7HFP5		D	S	R	R	T	S	D	G	R	A	L
K. pneumoniae	Q9F435		D	S	R	R	T	S	D	G	R	A	L
S. meliloti	Q92SD2	*Clr*	T	R	R	A	T	T	D	A	R	A	L
R. centenum	B6IXV7	*CgrA*	S	N	R	R	T	S	N	R	R	A	L
M. magnetotacticum	Q2W4U7		S	G	R	D	A	D	S	S	R	A	L
P. aeruginosa	Q9I6L5		S	I	R	K	A	F	P	Q	R	A	L

G cluster

FIG S5 Differences for sequence motifs in the cNMP binding site and the hinge/α5 and α6 regions. Several mutations in these regions as indicated below the *E. coli* CAP sequence render CAP cGMP-sensitive (a: Yoon *et al.*, Bacteriol 190, 4532–4540 (2008); b: Passner *et al.*, J Mol Biol. 304, 847-59 (2000); c: Yoon *et al.*, J Biol Chem 282, 3632–3639 (2007)). Almost all of these residues are conserved in the main Crp cluster, whereas the proteins from cluster G like Clr show higher diversity.
