# Supplementary material for: Structural Basis of Dual Specificity of Sinorhizobium meliloti Clr, a cAMP and cGMP Receptor Protein
Source: mBio. 2023 Apr 5;14(2):e03028-22. doi: 10.1128/mbio.03028-22 (PMC10127613; doi:10.1128/mbio.03028-22)
Supplement: FIG S4 [file mbio.03028-22-s0004.pdf]

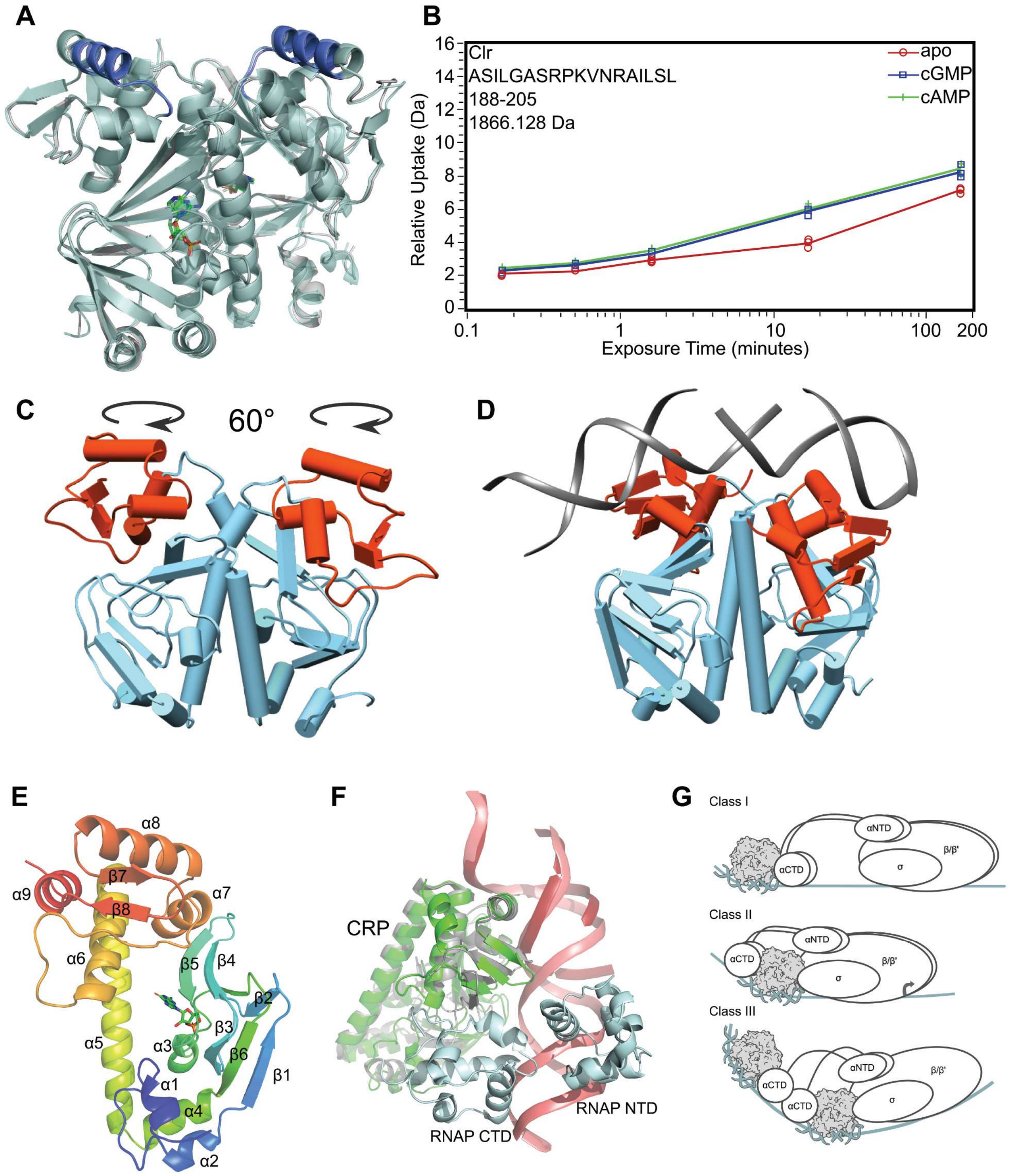

FIG S4 Structural features of DNA binding by Clr. Nucleotide binding triggers conformational transition in HTH DNA binding domain of Clr: (A) Localization of the peptide shown in (B) in the cAMP (grey) and cGMP (teal) bound structure. (B) Deuterium uptake increases upon nucleotide addition, as the movement in helix 8 results in increased solvent accessibility. (C) Model of Clr in the unliganded state based on a CAP NMR structure (PDB-ID: 2WC2). Helix 8 is rotated by 60° in comparison to (D), where the helix is inserted in the DNA major groove. Structural analysis of DNA bound Clr structure: (E) Secondary structure nomenclature of cAMP bound Clr monomer as used in this publication. Helix α1-α4 and strands β1-β6 for the cNMP binding domain in a beta-barrel fold, α5 is the zipper-like dimerization interface, whereas α6-α9 and β7 and 8 are part of the HTH DNA binding domain. (F) Structural overlay of a DNA-bound Clr monomer with CTD and NTD of the RNA polymerase of the *E. coli* class II transcription activation complex (PDB-ID: 6PB6). The interface shows slight deviations; however, we were clearly able to show, that Clr is able to interact with *E. coli* RNAP. (G) Promoter architectures differ in the assembly sequence of transcription activation components. In Class I promoters the CRP interacts with the CTD of the RNAP only, whereas in class II promoters the CRP is enclosed by the RNAP. Class III promoters involve two CRP binding sites.
